# Targeted mindfulness and self-compassion improve long-term stress reduction in distance learning students: a randomized trial

**DOI:** 10.3389/fpsyg.2025.1678094

**Published:** 2026-01-14

**Authors:** Lotte Bock, Miriam Hägerbäumer, Erik Riedel, Madiha Rana

**Affiliations:** 1 Department of Psychology and Self-Regulation, Leuphana University of Lüneburg, Lüneburg, Germany; 2 Department of Psychology, University of Applied Sciences EURO-FH in Hamburg, Hamburg, Germany

**Keywords:** mindfulness, self-compassion, gratitude, perceived stress, distance learning, digital mental health, emotional resilience, cognitive load theory

## Abstract

**Introduction:**

Stress remains a critical barrier to psychological wellbeing and academic functioning among university students, particularly in cognitively demanding distance learning contexts. These students often experience role conflict, social isolation, and increased mental load, which can hinder effective learning. This study examined whether a targeted web-based mindfulness intervention—focused on self-compassion and gratitude—produces more sustainable psychological benefits than a comprehensive mindfulness program.

**Methods:**

This randomized controlled trial included 167 university students who were randomized to one of two intervention arms; the final analytic sample comprised 98 participants after pre-specified, arm-blind outlier handling and exclusions due to missingness. Both interventions were delivered over 28 days using daily digital exercises. Measures of mindfulness, self-compassion, and perceived stress were collected at baseline, post-intervention, and at a three-month follow-up.

**Results:**

Both intervention groups showed significant short-term improvements in mindfulness and perceived stress (*p* < 0.001). However, only participants in the targeted intervention group sustained reductions in perceived stress at the three-month follow-up (*p* < 0.01), with T3 maintenance assessed within arms.

**Discussion:**

The targeted program was intentionally structured using cognitive learning theories—specifically Cognitive Load Theory to reduce extraneous processing and Spaced Repetition Theory to support memory consolidation—by emphasizing repetition of two core practices. The findings suggest that simplified, theory-informed mindfulness interventions may improve psychological resilience in cognitively burdened learners by facilitating deeper internalization and emotional regulation. This study contributes to educational psychology by demonstrating how instructional design grounded in cognitive theory can enhance mental health and support learners’ capacity to manage stress in demanding academic contexts.

## 1 Introduction

Stress is a well-documented challenge affecting students’ wellbeing and academic performance (El Ansari and Stock, 2010). The rise of digital learning environments, accelerated by the COVID-19 pandemic, has expanded the number of distance learners worldwide (Zawacki-Richter, 2021). These students face unique challenges such as reduced interaction with peers and instructors, which necessitates greater self-discipline (Giusti et al., 2021; Lamanauskas and Makarskaitė-Petkevičienė, 2021; Zinchenko and Gusarenko, 2022). While online learning offers flexibility, many distance learners juggle multiple roles—employee, parent, and student—further exacerbating stress, especially in the absence of peer support (Giusti et al., 2021; Lischer et al., 2022).

Student stress manifests in anxiety, depression, and feelings of overwhelm, which impair academic performance and increase risks of sleep disorders, substance use, and burnout (Bayram and Bilgel, 2008; Deng et al., 2022; Shaffique et al., 2020; Buenconsejo et al., 2024). A meta-study by Pascoe et al. (2020) showed that stress undermines cognitive functioning, while Lipson et al. (2015) linked unmanaged stress to lower grades and higher dropout rates. Distance learners are particularly vulnerable due to reduced peer interaction and greater reliance on digital resources (Fawaz and Samaha, 2021). A survey of 5,721 German distance learners found significantly elevated stress levels driven by competing academic, professional, and personal demands (Apolinário-Hagen et al., 2017), underscoring the need for tailored and accessible interventions (Lischer et al., 2022).

Graduate students, including those pursuing master’s and doctoral degrees, represent a subgroup of learners exposed to particularly high psychological demands. Intense academic workloads, career uncertainty, financial pressures, and limited support networks place them at elevated risk for stress, burnout, and attrition (Peltonen et al., 2017). Similar to other distance learners, they also face challenges such as reduced peer interaction and reliance on digital platforms, which can further exacerbate mental health concerns (Ludwig et al., 2020). Although the present study examines distance learners more broadly, its findings may be especially relevant for graduate students, as interventions that foster resilience, self-compassion, and sustained wellbeing could help mitigate dropout risk and support healthier academic trajectories. In line with broader educational shifts such as Education 5.0, which emphasizes personalized learning experiences that account for mental health and wellbeing (Ahmad et al., 2023), the present study explores accessible, web-based interventions that support resilience and emotional balance among distance learners.

Mindfulness-based approaches provide one such avenue. Mindfulness can help students cultivate awareness of stressors and adopt adaptive coping strategies (Dawson et al., 2020; Miyoshi, 2019; Yuan, 2022). Self-compassion fosters kinder self-responses in the face of inadequacy or overwhelm, while gratitude shifts attention toward positive experiences, enhancing emotional balance and social connectedness (Smeets et al., 2014; Ewert et al., 2021; Cregg and Cheavens, 2021; Davis et al., 2016). Collectively, these practices may be especially beneficial for distance and graduate learners, who often lack immediate peer support (Hefner and Eisenberg, 2009).

Several internet-based stress-management programs in Germany, such as the StudiCare Stress Program (Kählke et al., 2019) and (HelloBetter Stress und Burnout, n.d.), have been evaluated and shown to be effective. However, they primarily provide general coping strategies and do not compare targeted versus comprehensive approaches or explicitly apply learning theories such as Cognitive Load Theory (CLT; Sweller, 1988, 2020; Ouwehand et al., 2025) or Spaced Repetition Theory (SRT; Cepeda et al., 2008).

The present study examines whether mindfulness, self-compassion, and gratitude can enhance resilience, stress management, and wellbeing among distance learners. We compare two 28-day interventions: a comprehensive mindfulness program (*Mind2Full*) and a targeted program focusing only on self-compassion and gratitude (*ComGrat*). The *ComGrat* program leverages CLT to minimize extraneous cognitive load and SRT to strengthen memory retention through consistent practice. By reducing unnecessary complexity while embedding core emotional skills, *ComGrat* offers a cognitively accessible and sustainable approach. Exploring how such targeted interventions compare with comprehensive programs may provide valuable insights into tailoring psychological support for diverse learner populations.

### 1.1 Targeted vs. comprehensive mindfulness: the roles of self-compassion and gratitude

Mindfulness-Based interventions (MBIs) are widely recognized for their effectiveness in reducing stress and improving psychological wellbeing (Miyoshi, 2019; Dawson et al., 2020; Yuan, 2022). MBIs are structured programs designed to cultivate mindfulness—a state of present-moment awareness and nonjudgmental acceptance of thoughts, feelings, and bodily sensations—through practices such as meditation, mindful breathing, and body scans (Kabat-Zinn, 2005; Zuo et al., 2023; Halladay et al., 2019; Sperling et al., 2023; Wang et al., 2024; Randhawa and Vella-Brodrick, 2025). Meta-analyses have consistently demonstrated that both structured and self-directed MBIs can alleviate symptoms of stress, anxiety, and depression while enhancing academic performance among student populations (Flinchbaugh et al., 2012; Herizchi et al., 2016; Halladay et al., 2019; Shapiro et al., 1998; Bamber and Kraenzle Schneider, 2016; Zou et al., 2023; Gu et al., 2015; González-Martín et al., 2023; Shapiro et al., 2005). Typically spanning 4–8 weeks, these programs involve daily mindfulness practices ranging from 10 to 45 min. However, most MBIs integrate a wide variety of mindfulness techniques, such as meditation, body scans, and mindful movement (Cavanagh et al., 2014; Spijkerman et al., 2016; Bohlmeijer et al., 2010), which can make it challenging to pinpoint the specific contributions of individual components like self-compassion and gratitude.

Self-compassion and gratitude, often included within broader mindfulness frameworks, have been independently shown to reduce stress and improve psychological wellbeing (Buenconsejo et al., 2024; Smeets et al., 2014). Meta-analyses on self-compassion (Ewert et al., 2021; Kirby et al., 2017; Ferrari et al., 2019) and gratitude interventions (Cregg and Cheavens, 2021; Davis et al., 2016; Dickens, 2017) have confirmed their ability to decrease depression, anxiety, and self-criticism while enhancing resilience and emotional wellbeing. Research has also highlighted the benefits of self-compassion for improving coping mechanisms during stressful situations, including online learning contexts (Chen et al., 2022). Similarly, gratitude practices have been associated with increased positive affect and improved interpersonal relationships, which are particularly beneficial in mitigating the isolation often experienced by distance learners (Cregg and Cheavens, 2021). Despite this evidence, studies explicitly examining the combined effects of self-compassion and gratitude remain scarce, particularly for distance learners who face unique stressors such as role conflict, social isolation, and reduced access to support systems.

This study addresses this gap by directly comparing a comprehensive mindfulness program (*Mind2Full*) with a targeted intervention focusing solely on self-compassion and gratitude (*ComGrat*). This comparison builds upon prior research by isolating the effects of these two constructs, allowing for a more precise evaluation of their combined impact. While previous studies have evaluated the efficacy of broader mindfulness programs, few have systematically investigated whether targeted interventions might yield greater benefits for specific populations, such as distance learners. For instance, a comparison of *Mindfulness-Based Stress Reduction (MBSR)* and *Mindful Self-Compassion (MSC)* programs found that *MSC* resulted in greater improvements in self-compassion and emotional resilience, despite both programs effectively reducing stress (Ferrari et al., 2019; Neff and Germer, 2021). Similarly, meta-analyses of gratitude-focused interventions have highlighted their capacity to enhance wellbeing and reduce symptoms of depression and anxiety, often exceeding the effects of broader mindfulness practices (Cregg and Cheavens, 2021). By focusing on the specific constructs of self-compassion and gratitude, the *ComGrat* intervention aims to address the stressors unique to distance learners more effectively than a general mindfulness approach. This study builds on recent findings suggesting that targeted interventions may not only enhance psychological outcomes by reducing cognitive load but also foster deeper engagement and more sustainable long-term benefits.

### 1.2 Theoretical basis for the interventions

The study is grounded on the *Cognitive Load Theory* (CLT) (Sweller, 1988, seminal; Sweller, 2020; Ouwehand et al., 2025) and the *Spaced Repetition Theory* (SRT) (Cepeda et al., 2008) (see Table 1). CLT suggests that the human mind has a finite capacity for processing information. By carefully designing intervention materials to minimize extraneous cognitive load, unnecessary elements or distractions, such as overly complex instructions, unrelated theoretical concepts, or extraneous visuals, were excluded. Instead, materials were structured to focus solely on the core elements of self-compassion and gratitude. Each exercise was presented in simple, clear language to ensure participants could fully engage with the practices without cognitive overload (Sweller, 2010; Kalyuga, 2011). The *ComGrat* intervention, which emphasizes self-compassion and gratitude, minimizes unnecessary cognitive demands by narrowing its focus to two core mindfulness practices. This deliberate simplification aligns with *CLT*’s recommendation to reduce split attention and avoid integrating unrelated or competing tasks that may distract from the primary learning goals. This streamlined approach reduces distractions from learning multiple techniques simultaneously and aligns with evidence that a reduced cognitive load facilitates greater depth of processing and better retention of core content (Paas and Ayres, 2014). In this study, the application of *CLT* supports the hypothesis that participants in the *ComGrat* group will experience more effective engagement and learning compared to those in a broader mindfulness program, such as *Mind2Full*, which introduces new exercises daily. By focusing on only two components, participants can internalize the concepts at their own pace, accommodating individuals with varying levels of cognitive resilience. This adaptability enhances the intervention’s efficacy in heterogeneous groups (van Merriënboer and Sweller, 2005).

**Table 1:** Mapping of theoretical frameworks to intervention design components.

Theoretical concept	Design principle	Description	ComGrat intervention	Mind2Full intervention
Cognitive load theory (CLT)	Minimize extraneous cognitive load	Reduce complexity	Focuses on two core practices (self-compassion and gratitude)	Introduces a new mindfulness exercise each day
Cognitive load theory (CLT)	Simplify and structure materials	Deliver content clearly	Daily exercises delivered in a consistent format	Varied instructions and techniques
Cognitive load theory (CLT)	Avoid split attention and distractions	Reduce cognitive strain	Repeats same practices to focus engagement	Multiple simultaneous practices may increase load
Spaced repetition theory (SRT)	Reinforce learning through daily repetition	Enhance long-term retention	Repeats same two exercises daily for 28 days	Practices rotate daily
Spaced repetition theory (SRT)	Encourage automation	Build habits	Promotes automatic coping skills	Limited automation due to content variety

This table illustrates how Cognitive Load Theory (CLT) and Spaced Repetition Theory (SRT) informed the design of the two mindfulness interventions. The *ComGrat* intervention employed focused content and daily repetition, while *Mind2Full* incorporated a broader range of techniques with greater variation.

Additionally, *SRT* suggests that repeated exposure to the same material over time enhances long-term retention. In the *ComGrat* intervention, repetition was operationalized by delivering the same two core mindfulness exercises—one focused on self-compassion and the other on gratitude—every day for 28 days. These exercises were carefully timed to balance engagement and reinforcement, ensuring participants could revisit and internalize these practices consistently. The daily repetition was supplemented with reflective prompts, which were designed to strengthen the emotional resonance and cognitive connection to the core practices. This approach aligns with *SRT*’s principles of distributing learning opportunities to avoid cognitive fatigue while promoting consolidation over time (Cepeda et al., 2006; Kang, 2016). Research on *SRT* has demonstrated that revisiting core practices through consistent repetition strengthens memory consolidation and promotes the automation of skills (Cepeda et al., 2006; Kang, 2016). Unlike the *Mind2Full* course, which introduces a variety of new mindfulness exercises daily, the *ComGrat* intervention repeats the same core exercises over the 28-day program. This repetition reinforces self-compassion and gratitude, promoting the long-term internalization of these practices. Evidence from studies on repetitive learning supports the hypothesis that this design will result in deeper skill consolidation and more sustainable emotional benefits (Rawson and Dunlosky, 2011; Carpenter et al., 2012).

The integration of *CLT* and *SRT* provides a comprehensive framework for understanding the design and predicted effectiveness of the *ComGrat* intervention. While *CLT* explains how reducing extraneous cognitive load allows participants to engage more deeply with self-compassion and gratitude, *SRT* highlights the importance of timing and structured repetition, ensuring participants have consistent opportunities to revisit and reinforce these practices. By carefully calibrating the frequency and timing of the exercises, the intervention was designed to optimize memory retention while minimizing the risk of cognitive fatigue. Together, these theories suggest that *ComGrat* optimizes both immediate and long-term outcomes by combining cognitive efficiency with memory consolidation. This framework justifies the hypothesis that the targeted, repetitive nature of *ComGrat* will yield superior reductions in perceived stress and longer-lasting emotional resilience compared to the more generalized *Mind2Full* approach.

In line with these theoretical underpinnings, both interventions were structured to last 28 consecutive days, with participants engaging in manageable daily exercises. The *Mind2Full* intervention, based on the principles of Füge, “Mindfulness-Based Stress Reduction (MBSR) Authorized Curriculum Guide, n.d.” to the existing reference (Kabat-Zinn, 2003), offers a diverse array of mindfulness practices, fostering general mindfulness skills. In contrast, the *ComGrat* intervention combines elements of Mindfulness-Based Compassion Learning (MBCL) (Dickens, 2017) and Mindful Self-Compassion (MSC) (Van Den Brink and Koster, 2015), along with a focused gratitude intervention (Neff and Germer, 2013; Seligman et al., 2005; Wood et al., 2010). This targeted approach leverages the combined strengths of *CLT* and *SRT* to reduce cognitive load while reinforcing self-compassion and gratitude through daily repetition. By facilitating deeper emotional engagement and sustained practice, this design aligns with evidence that targeted, repeated interventions are particularly effective in reducing perceived stress and promoting emotional wellbeing over time (Davis et al., 2021).

### 1.3 Hypothesis

The objective of this study is to examine the immediate (T2) and long-term (T3) impacts of two 4-week self-directed web-based mindfulness interventions on the mindful-ness skills, self-compassion skills, and subjective perception of stressors in distance learning students. To investigate this question, two experimental groups (EG) and one control group (CG) were subjected to testing prior to the intervention (T1), immediately after the interventions (T2), and 3 months after the interventions ended (T3). The two experimental groups (EGs), designated A and B, each participated in a four-week self-directed web-based intervention. Group A was subjected to the comprehensive mindfulness intervention, *Mind2Full*, while Group B was subjected to the self-compassion and gratitude intervention, *ComGrat*. The study proposed three hypotheses to assess the short- and long-term effects of the interventions on mindfulness skills, self-compassion, and stress perception.

#### 1.3.1 Hypothesis 1: mindfulness skills

*H1a*: Participants in both experimental groups (*Mind2Full* and *ComGrat*) will show a significant improvement in mindfulness skills immediately after the intervention (T2) compared to the control group.

*H1b*: Participants in the *Mind2Full* group will show a greater improvement in mindfulness skills at T2 than participants in the *ComGrat* group, due to the exposure to all mindfulness skills.

*H1c*: Both experimental groups will maintain a significant improvement in mindfulness skills 3 months after the intervention (T3), with the *Mind2Full* group expected to show stronger long-term effects due to the comprehensive nature of the intervention.

#### 1.3.2 Hypothesis 2: self-compassion

*H2a*: Participants in both experimental groups (*Mind2Full* and *ComGrat*) will exhibit a significant increase in self-compassion immediately after the intervention (T2) compared to the control group.

*H2b*: Participants in the *ComGrat* group will show a significantly greater improvement in self-compassion at T2 than those in the *Mind2Full* group, as this intervention specifically focuses on self-compassion and gratitude.

*H2c*: The *ComGrat* group will exhibit significantly higher levels of self-compassion 3 months after the intervention (T3) compared to the *Mind2Full* group, as the focused nature of the intervention is expected to have a stronger long-term impact.

#### 1.3.3 Hypothesis 3: perceived stress

*H3a*: Participants in both experimental groups (*Mind2Full* and *ComGrat*) will report a significant reduction in perceived stress immediately after the intervention (T2) compared to the control group.

*H3b*: The reduction in perceived stress will be similarly strong in both experimental groups at T2, as both interventions contain mindfulness elements that target stress reduction.

*H3c*: The *ComGrat* group will show a significantly greater reduction in perceived stress 3 months after the intervention (T3) compared to the *Mind2Full* group, due to the long-term effects of the focused intervention.

## 2 Materials and methods

The study was approved by the ethics committee of the European University of Applied Sciences (EKEFH02/22), and all participants provided written informed consent. A randomized controlled trial (RCT) design was employed, with assessments conducted at three time points: baseline (T1), immediately after the intervention (T2), and 3 months post-intervention (T3). The study was conducted between 4 February and 17 June 2023.

Participants were recruited via a centralized email distributed to all active distance-learning students at the European University of Applied Sciences through their institutional student accounts. The recruitment email detailed the study’s objectives, procedures, and eligibility criteria, emphasizing the voluntary nature of participation. Students enrolled in bachelor’s or master’s degree programs with internet access were eligible to participate. No additional exclusion criteria were applied, meaning the study employed convenience sampling from the accessible student population rather than random sampling. As part of the informed consent process, participants were instructed to assess their own physical and mental fitness and to refrain from yoga or stretching exercises if they experienced any limitations.

After completing the baseline assessment (T1), participants were randomly assigned to one of three groups—the comprehensive mindfulness program (*Mind2Full*), the self-compassion and gratitude intervention (*ComGrat*), or the waitlist control—using a computerized randomization procedure. Random assignment ensured that each participant had an equal probability of allocation to any of the groups, thereby supporting internal validity.

The sample size calculation was conducted with G*Power 3.1 (Faul et al., 2007) for a repeated-measures ANOVA (within–between interaction; 3 groups × 3 time points). We assumed f=0.25 (≈ $\eta_{p}^{2}\approx 0.059$ ), α=0.05 , 1−β=0.95 , correlation among repeated measures = 0.50, and ε=1 . Under these assumptions, the minimum required total sample size was N=54 . The choice of f=0.25 was informed by meta-analyses in student samples of web-based mindfulness, self-compassion, and gratitude interventions, which typically report small-to-moderate effects (e.g., Bamber and Kraenzle Schneider, 2016; Ewert et al., 2021; Cregg and Cheavens, 2021; Wright et al., 2023). In addition, the repeated-measures design increases power by reducing within-subject variability. Based on this literature, we considered a time × group interaction of f=0.20 (≈ $\eta_{p}^{2}\approx 0.038$ ) on perceived stress (PSQ) to be the smallest effect of practical relevance. Balancing feasibility and expected attrition, our *a priori* power analysis targeted f=0.25 for the 3 × 3 interaction ( α=0.05 , 1−β=0.95 ). Multiplicity control. We specified a hierarchical (gatekeeping) sequence at the endpoint level: PSQ (primary) → CHIME (key secondary) → SCS (key secondary). We formally tested the next endpoint only if the preceding time × group interaction was significant at α=0.05 . Within any endpoint that passed the omnibus test, pairwise post-hoc contrasts were Tukey-adjusted. All other analyses (e.g., subgroup/mechanistic probes) are labelled exploratory and reported with exact *p*-values and 95% CIs without additional family-wise adjustment. This approach constrains Type-I error for confirmatory claims while maintaining transparency about secondary findings.

Following data cleaning procedures—including outlier removal and exclusion due to missing data—the final analytic sample comprised approximately 30 participants per group (*N* = 98 total). While this reduction still permits detection of within-subject effects in a repeated-measures design, we acknowledge reduced power for small between-group effects. Outliers were flagged using two *a priori*, group-agnostic rules applied uniformly across outcomes and time points before any group comparisons: (i) standardized scores with |*Z*| ≥ 3 and (ii) values outside 1.5 × IQR. The three-sigma rule targets extreme values under approximately normal sampling, whereas the Tukey 1.5 × IQR rule is non-parametric and robust to skew/heavy tails; using both reduces reliance on a single distributional assumption. Randomization was preserved throughout, and included vs. excluded participants did not differ systematically on baseline sociodemographic or psychological measures. To assess robustness, we (a) re-estimated all models including the previously excluded cases, with the overall pattern of results unchanged though some effect sizes attenuated (Table 2), and (b) fitted intention-to-treat linear mixed-effects models using maximum likelihood to all randomized participants and all available observations; these converged on the same substantive conclusions (results summarized in the Results section).

**Table 2:** Sensitivity analyses of main outcomes with vs. without outlier exclusion.

Outcome variable	Pattern with outlier exclusion	Pattern without outlier exclusion
Mindfulness (CHIME)	Significant within-group improvements in both intervention groups at T2; *ComGrat* showed stronger long-term gains at T3	Same overall pattern; effect sizes attenuated
Self-compassion (SCS)	Significant within-group improvements at T2 in both groups; *ComGrat* retained modest long-term benefit at T3	Same overall pattern; effects slightly smaller
Perceived stress (PSQ)	Significant short-term reductions in both interventions at T2; *ComGrat* showed more durable long-term effects at T3	Same overall pattern; long-term differences less pronounced

Outliers were identified using two pre-specified criteria (|*Z*| ≥ 3 and values outside 1.5 × IQR), applied uniformly across outcomes and time points before any group comparisons; group assignment was not considered during this process. Randomi**z**ation was preserved, and included vs. excluded participants did not differ systematically on baseline sociodemographic or psychological measures. “Full sample incl. Outliers” re-estimates the same complete-case RM-ANOVA models without outlier exclusions; the overall pattern of results remained, with some attenuation of effect sizes. As an additional robustness check, intention-to-treat linear mixed-effects models using all available observations reached the same substantive conclusions (see Results: ITT mixed-effects summary).

### 2.1 Format and content of the interventions

The *Mind2Full* and *ComGrat* interventions were designed with a similar methodology. The interventions were conducted over a 28-day period and were disseminated automatically via email using a web-based campaign system tool. Each intervention comprised exercises that collectively amounted to 15–20 min of daily practice. The *Mind2Full* and *ComGrat* interventions were designed, developed, implemented, and conducted by a psychologist and certified mindfulness trainer, meditation teacher, and stress management coach. Participant adherence to the intervention was indirectly monitored through tracking email engagement, such as how often the intervention emails were clicked or opened. However, as this metric does not confirm whether participants completed the exercises, it has not been reported in the study. Instead, we relied on participants’ signed commitment during registration, in which they expressed their willingness to dedicate 20 min per day to the intervention exercises. Examples of the intervention emails and psychoeducational materials can be found in Supplementary File 1.

#### 2.1.1 Mindfulness intervention: *Mind2Full*

The *Mind2Full* intervention comprised 28 units, in addition to an introductory unit. Each morning between 6:00 and 7:00 a.m., participants received a daily email containing three videos: a mindfulness impulse, office yoga exercises, and meditation. The content of the mindfulness impulses was diverse, comprising a range of practical exercises. These techniques included mindful breathing, mindful eating, awareness of thoughts, emotional regulation strategies and grounding techniques. The objective was to provide participants with the capacity to integrate these techniques into their daily routines. To illustrate, a mindfulness impulse might comprise a brief exercise on mindful walking, encouraging participants to focus on the sensation of each step and the environment around them, or an encouragement to record feelings and thoughts throughout the day. The office yoga exercises were designed in such a way that they could be performed by any individual, regardless of their level of experience or access to specialized equipment. The exercises focused on gentle stretching, including neck rolls, seated twists, and shoulder shrugs. These could be performed on a chair at home or in the workplace. The objective was to reduce physical tension and encourage relaxation, even in a restricted work environment. For instance, participants may be instructed in the performance of a fundamental seated forward bend, which serves to relieve muscular tension in the lumbar and shoulder regions. The meditations were adaptable, allowing participants to engage in the practice in any comfortable position or space. The meditations comprised a series of guided visualizations, body scans and breath awareness practices. To illustrate, a typical meditation session might comprise a guided body scan, whereby participants direct their attention to each part of their body, identifying any areas of tension and consciously relaxing those regions. The adaptability of the meditation practices permitted participants to engage in the sessions in a manner that was consistent with their individual preferences and circumstances. The introductory unit provided an overview of the course structure, a general introduction to mindfulness, and practical advice for successfully completing the course. The course encompassed eight fundamental elements of the Mindfulness-Based Stress Reduction (MBSR) programmeme. The eight components of the course were as follows: (1) awareness of inner experience, (2) awareness of outer experience, (3) conscious action in the present, (4) an accepting, non-judgmental, self-compassionate, grateful attitude, (5) a non-reactive, decentered orientation, (6) an open, non-avoidant attitude, (7) relativity, and (8) insightful understanding (Bergomi et al., 2013b). This comprehensive design ensured that participants could readily incorporate mindfulness into their daily routines, irrespective of their prior experience or current circumstances.

The intervention was theoretically grounded in the standard MBSR curriculum (*Mindfulness-Based Stress Reduction (MBSR) Authorized Curriculum Guide*) and aligns with the transactional model of stress, which emphasizes the critical role of personal perception and appraisal in stress and coping (Kavanagh, 1986). The intervention also fostered the development of personal resources, including self-efficacy (Hobfoll, 1989), a mindful and stress-reducing interpretation of events, a compassionate and accepting attitude towards oneself, the ability to detach from thoughts and emotions, and the capacity to maintain emotional balance (Le Fevre et al., 2003).

#### 2.1.2 Self-compassion and gratitude intervention: *ComGrat*

The second four-week web-based intervention was centered on the themes of self-compassion and gratitude, building on two of the components also found in the *Mind2Full* intervention. The objective of the intervention was to cultivate the development of self-compassion and gratitude, encompassing non-judgmental awareness, self-self-compassion, recognizing shared humanity, applying mindfulness in challenging situations, and empathy-enhancing exercises (Neff K., 2003; Neff K. D., 2003). This was achieved through the engagement in *loving-kindness meditations* (*LKM*) (Shonin et al., 2015) and the maintenance of a gratitude journal (Fritson, 2008). *LKM* is a systematic practice that involves directing feelings of kindness and compassion towards oneself, loved ones, neutral individuals, and even those with whom one has conflicts. For example, participants may recite phrases such as “May I be happy, may I be healthy,” gradually extending these aspirations to others. In the initial two-week period, participants were introduced to the various forms of this meditation, including focusing on specific individuals or broadening the focus to larger groups. Subsequently, participants were encouraged to select and practice the form that was most resonant with them.

Gratitude journaling entailed a daily reflection on aspects of life for which participants expressed gratitude. It was recommended that participants maintain a daily journal, either digitally or manually, and engage in a reflection on gratitude in the evening. The guidelines recommended the listing of three to five specific instances per day, such as a positive interaction, a moment of beauty, or a minor accomplishment. To illustrate, a participant might record a gratitude-related experience such as, “Today, I am grateful for the warm cup of coffee that energized my morning,” or “I appreciate the support I received from my colleague.” The objective of this practice was to alter the focus of attention from sources of stress to positive experiences, thereby reinforcing a mindset of appreciation and resilience. Meta-studies have demonstrated the positive psychological effects of gratitude journaling (Smyth, 1998; Sohal et al., 2022). This technique, which gained popularity in the early 2000s, entails individuals regularly recording the things for which they are thankful. Gratitude journaling is a widely utilized technique in positive psychology (Seligman et al., 2005). It helps individuals shift their focus from perceived deficiencies to acknowledging and appreciating existing blessings, thereby fostering a more optimistic perspective. The available evidence suggests that maintaining a gratitude journal can facilitate the development of humility (Kruse et al., 2014) and enhance overall wellbeing (Killen and Macaskill, 2015; Dickens, 2017).

The participants were provided with a daily email comprising a *LKM*, instructions for the meditation, and a prompt to reflect on their gratitude entries. Moreover, the daily emails incorporated a psychoeducational element. The psychoeducational content was designed to facilitate a more profound comprehension of self-compassion and gratitude. To illustrate, one email might elucidate the concept of self-compassion by elucidating how it entails treating oneself with the same benevolence and comprehension as one would extend to a friend in need. Another email might examine the advantages of gratitude, underscoring research indicating that the regular acknowledgement and appreciation of favorable aspects of life can enhance overall wellbeing. The aforementioned emails served the dual purpose of pedagogical instruments and subtle prompts, encouraging the integration of these concepts into daily life through the practice of meditation and journaling.

Both interventions place an emphasis on meditation, which has been demonstrated to confer a multitude of benefits in numerous studies (Le Fevre et al., 2003). This highlights its efficacy in mitigating stress and anxiety, as well as enhancing overall mental wellbeing. It has been demonstrated that the practice of meditation can also exert a beneficial effect on personal self-compassion skills (Golden et al., 2021). A meta-analysis conducted by Wilson et al. (2019) indicates that self-compassion-related therapies are generally effective in improving psychological outcomes, such as reducing depression, anxiety, and stress, while increasing wellbeing and life satisfaction. A meta-analysis conducted by Zeng et al. (2015) focusing on the *LKM* revealed that the *LKM* has a significant positive effect on self-compassion, along with other psychological benefits, including an increase in positive emotions and a reduction in negative emotions. It can be reasonably inferred that the practice of loving-kindness meditation may prove an efficacious strategy for enhancing emotional wellbeing and fostering a more benevolent disposition among distance learners. In conclusion, the evidence suggests that meditation is an effective method for alleviating anxiety and stress. The *LKM*, in particular, has been shown to be beneficial for fostering a kind and caring approach.

In conclusion, the *Mind2Full* was a comprehensive, self-directed online intervention encompassing the full spectrum of mindfulness, whereas *ComGrat* was a concentrated, self-directed online intervention focusing on two pivotal aspects of mindfulness: self-compassion and gratitude. The effects of mindfulness training, self-compassion training and gratitude training on stress have been separately examined in research studies. The combination of self-compassion and gratitude is now to be examined on the hypothesis that a focused approach might result in even stronger positive effects due to the smaller cognitive load in comparison to standard comprehensive mindfulness approaches.

### 2.2 Questionnaires

In order to test this hypothesis, three questionnaires were administered: the CHIME, the PSQ, and the SCS. The selection was guided by their ability to capture core cognitive and emotional outcomes aligned with the intervention’s theoretical underpinnings. Specifically, the CHIME (Comprehensive Inventory of Mindfulness Experiences) reflects increased attentional regulation and emotional awareness, which are expected outcomes of mindfulness practice and reduced cognitive overload (CLT) (Dawson et al., 2020; Sweller, 2020). The PSQ (Perceived Stress Questionnaire) captures changes in subjective stress appraisal, which may decrease as cognitive load is minimized and emotion regulation skills improve through repetitive training (CLT and SRT). The SCS (Self-Compassion Scale) assesses self-kindness, emotional resilience, and reduced self-criticism—core targets of the *ComGrat* intervention—and is particularly suited to detect skill acquisition resulting from structured, repeated practice as theorized in SRT. Together, these tools offer a robust measure of the key outcomes the interventions aimed to produce through reduced complexity (CLT) and enhanced repetition (SRT).

Participants completed pre- and post-intervention self-assessment questionnaires (T1, T2, and T3) online to measure mindfulness skills, perceived stress, and perceived levels of self-compassion and gratitude, respectively. To ensure the authenticity and independence of responses, participants were required to set up a unique password during registration. This password was created using a combination of their mother’s name, father’s name, birth city, and birthday, ensuring it was both secure and personal. Participants used this password for identification each time they accessed the questionnaires, preventing unauthorized access and maintaining data integrity. These measures ensured that all responses could be reliably attributed to the intended participants, while safeguarding their privacy.

#### 2.2.1 CHIME—comprehensive inventory of mindfulness experiences

The mindfulness skills in this study were assessed using the Comprehensive Inventory of Mindfulness Experiences (*CHIME*) questionnaire developed by Bergomi et al. (2013a). This tool was specifically designed to evaluate eight fundamental mindfulness skills, drawing from the constructs of eight existing mindfulness questionnaires (Bergomi et al., 2013b; Bergomi et al., 2014). The *CHIME* is unique in that it avoids using terminology that might be difficult for individuals without meditation experience to understand, making it accessible to a broader audience (Bergomi et al., 2014). The *CHIME* consists of 37 items presented as first-person statements, with respondents rating each item on a 6-point bipolar Likert scale ranging from ‘almost never’ to “almost always.” Following scoring guidelines, all items were scored such that higher values reflect greater mindfulness skills. No reverse coding was necessary. A total score was calculated by computing the average of all 37 items, in line with the standard scoring procedure.

The questionnaire is divided into eight subscales, each assessing a different dimension of mindfulness. The questionnaire assesses awareness of inner experiences, as indicated by items such as “I notice when my mood begins to change,” while it gauges awareness of outer experiences, as evidenced by statements such as “I am aware of sounds around me, like birds chirping or traffic noise.” The assessment of conscious action and presence is conducted through items such as “I find myself engaging in activities without paying attention.” Furthermore, the questionnaire assesses an accepting, non-judgmental, and self-compassionate attitude, as evidenced by statements such as “I am able to accept unpleasant feelings without attempting to alter them.” An objective and detached perspective is gauged by statements such as “I am able to observe my thoughts without becoming enmeshed in them,” while an open and non-avoidant stance is evaluated with items like “I am receptive to experiencing whatever thoughts and emotions arise.” The assessment of relativity is conducted through statements such as “I recognize that my thoughts are merely thoughts, not facts.” Conversely, the evaluation of insightful understanding is accomplished through items such as “I perceive the interconnectivity between my thoughts, feelings, and behaviors.” Each of these dimensions is essential for providing a comprehensive measure of mindfulness, reflecting a wide range of experiences and attitudes that contribute to mindful awareness and practice. The internal consistency of these subscales is robust, with Cronbach’s alpha values ranging from *α* = 0.70 to 0.86, indicating reliable measurement across the different facets of mindfulness (Bergomi et al., 2014). Although the CHIME includes eight subscales, only the total score was used in statistical analyses (e.g., ANOVA), as the study focused on overall changes in mindfulness rather than subscale-specific patterns.

The CHIME questionnaire has been validated extensively in adult populations, including those participating in psychological interventions and mindfulness-based training programs (Bergomi et al., 2013a; Bergomi et al., 2014). While specific studies validating CHIME among online learners are limited, its focus on universal mindfulness skills and its design to avoid meditation-specific terminology make it suitable for diverse populations, including adults engaged in online learning environments. Validation studies, such as Bergomi et al. (2013a, 2014) and Wilkinson et al. (2023), report robust psychometric properties across various contexts, with internal consistencies ranging from α = 0.86 to 0.94 across subscales, satisfactory test–retest reliability over 4 weeks (*r* = 0.72), and factorial validity confirmed through confirmatory factor analyses. A recent Rasch validation further demonstrated strong item fit and invariance across demographic subgroups (Wilkinson et al., 2023), reinforcing the scale’s applicability to the present study.

#### 2.2.2 PSQ—perceived stress questionnaire

The study employed the Perceived Stress Questionnaire (*PSQ*), developed by Fliege et al. (2005), as a measure of perceived stress. The German-language short version employed in this study comprises 20 items, distributed across four subscales: Worry, Tension, Joy, and Demands. Each subscale offers insight into the various ways in which individuals perceive, evaluate, and process stressful stimuli. To illustrate, the Worry subscale comprises items such as “You feel that your worries keep growing,” which reflect concerns about personal challenges. Items included in the Tension subscale may, for instance, reflect feelings of unease or stress-related tension, as indicated by the statement ‘You feel irritable.” The Joy subscale comprises items that reflect positive emotional experiences, such as “You feel full of energy,” which are inversely related to stress levels. The final subscale, entitled Demands, comprises items such as “You feel that too many demands are being placed on you,” which address the pressure exerted by external demands. Each item is rated on a 4-point Likert scale, ranging from “almost never” to “most of the time,” thereby providing a nuanced view of the frequency with which participants experience these feelings. The total PSQ was computed as the mean of all 20 items after reverse-scoring the negatively valenced subscales (Worry, Tension, Demands), so that higher scores indicate lower perceived stress.

The PSQ has demonstrated strong psychometric properties across multiple studies. Validation research reported Cronbach’s alpha values of *α* = 0.86 for the total score and α = 0.80–0.85 for subscales, indicating satisfactory internal consistency (Levenstein et al., 1993; Fliege et al., 2005). Split-half reliability has also been shown to be high, further confirming the robustness of the measure. Subscale scores were not analyzed separately in the present study due to our focus on overall stress levels. The PSQ has been widely validated in adult populations across occupational, educational, and clinical settings, with German-language short versions confirming its applicability to both working adults and students (Fliege et al., 2005; Kocalevent et al., 2007). These findings provide confidence in the PSQ’s suitability for assessing stress in the online learning population targeted in this study.

#### 2.2.3 SCS—self-compassion scale

The study employed the Self-Compassion Scale (SCS), developed by Neff K. (2003) and Neff K. D. (2003) and subsequently validated by Neff and Tóth-Király (2022), to assess levels of self-compassion and gratitude. The SCS is a 26-item self-report questionnaire utilizing a five-point Likert scale, ranging from “almost never” (1) to “almost always” (5), for the evaluation of various dimensions of self-compassion. These dimensions encompass self-kindness, common humanity, self-judgment, isolation, mindfulness, and the avoidance of over-identification. The construct of self-kindness is operationalized through items that assess the respondent’s capacity to demonstrate understanding and gentleness towards themselves during challenging circumstances. To illustrate, a typical item might be, “I endeavor to extend benevolence towards myself when I am experiencing emotional distress.” The concept of common humanity is evaluated through items that emphasize the recognition that personal struggles and failures are part of the shared human experience. For example, an item may state, “When I am feeling down, I remind myself that there are many other individuals in the world who are experiencing similar feelings.” The construct of self-judgment is operationalized through items that reflect harsh self-criticism, such as “I am disapproving and judgmental about my own flaws and inadequacies.” The construct of isolation is operationalized through items that reflect feelings of loneliness during challenging periods. An illustrative item is, “When I consider my shortcomings, it tends to engender a sense of detachment and isolation from the broader social context.” The SCS also incorporates components that assess mindfulness, such as maintaining a balanced perspective in the face of adversity. This is evaluated through items such as “When something painful happens, I try to take a balanced view of the situation.” Finally, avoiding over-identification with negative emotions or perceived failures is assessed through items such as “When I’m feeling down, I tend to obsess and fixate on everything that’s wrong.”

Reverse-coded items were adjusted in accordance with the published scoring key. The total score was computed as the average of all items after reverse coding, with higher scores reflecting greater self-compassion. Although the scale consists of six subscales, only the total score was used in this study’s analyses, as the primary research questions pertained to overall changes in self-compassion. The Self-Compassion Scale (SCS; Neff K., 2003; Neff K. D., 2003) has demonstrated excellent psychometric properties across a wide range of populations. Internal consistency is consistently high, with Cronbach’s alpha reported at α ≈ 0.92 for the total score, and subscales also showing satisfactory reliability (Neff K., 2003; Neff K. D., 2003; Neff and Tóth-Király, 2022). Test–retest reliability is likewise strong, with correlations up to r = 0.93 over three weeks (Neff K., 2003; Neff K. D., 2003). Multiple studies have confirmed the scale’s construct validity, factorial structure, and applicability across diverse adult samples, including students and individuals participating in online psychological interventions (Yarnell and Neff, 2013; Trompetter et al., 2017). While specific validation in distance-learning contexts remains limited, the emphasis on universal dimensions of self-compassion supports its relevance for the present study population.

### 2.3 Analytic approach

A total of 254 distance-learning students from the European University of Applied Sciences were invited to participate via email. Of these, 251 students registered for the study and provided informed consent. However, 84 students did not complete the baseline survey (T1), leaving 167 participants with baseline data. These participants were then randomly assigned to one of three groups using a computerized randomization procedure^1^: a comprehensive mindfulness intervention (*Mind2Full*; experimental group A (EG_A), *n* = 85), a self-compassion and gratitude intervention (*ComGrat*; experimental group B (EG_B), *n* = 82), or a waitlist control group (CG, *n* = 84). Random assignment ensured that each participant had an equal probability of allocation, thereby supporting internal validity. Sequence generation used a simple computer-generated list from Random.org with a 1:1:1 allocation ratio; no stratification or blocking was used. Allocation concealment was ensured by the survey platform’s automated randomization module, which released the assignment only after T1 submission; neither participants nor study staff could view or predict upcoming allocations. Accordingly, baseline (T1) measures were collected blind to assignment (self-administered online with no live assessors). Allocation concealment was maintained by an automated randomization script embedded in the online survey platform that released the assignment only after T1 submission; neither participants nor investigators could access or predict upcoming allocations.

To minimize bias, participants were only informed of their group assignment after completing the baseline questionnaire (T1); thus, both participants and study staff were blind to allocation at T1. All baseline measures were self-administered online with no live assessors. Post-allocation blinding of participants was not feasible due to the nature of the interventions. This ensured that their initial responses were not influenced by group knowledge, maintaining data integrity. Upon completing the first questionnaire, participants either received a link to register for their assigned intervention or were informed of their placement in the control group. Both experimental groups began the interventions simultaneously. The control group was informed they would gain access to the intervention after the second assessment (T2). Four weeks after T1, all participants completed the second questionnaire (T2). To assess long-term outcomes, participants from the original experimental groups completed a third questionnaire (T3), 3 months after the end of the interventions. The control group received access to the interventions immediately after the second assessment (T2) to maintain ethical standards and reduce dropout. As a result, their T3 data represent immediate post-intervention outcomes, equivalent to T2 for the experimental groups. These data were used to assess short-term effects within the waitlist group but were not included in long-term between-group comparisons, as the control group was no longer untreated at T3.

A mixed factorial repeated-measures ANOVA (3 groups × 3 time points) tested main effects of Time and Group and their interaction. Assumptions were evaluated on model residuals: residual histograms and Q–Q plots showed no material departures from normality; sphericity was tested with Mauchly’s test and Greenhouse–Geisser corrections applied where required; homogeneity of variances was examined with Levene’s tests. *Post-hoc* multiple comparisons used Tukey’s method with *α* = 0.05 (two-tailed).

Outliers were flagged using two *a priori*, group-agnostic rules applied uniformly across outcomes and time points before any group comparisons: (i) |*Z*| ≥ 3 and (ii) values outside 1.5 × IQR. The three-sigma rule targets extremes under approximate normality, whereas the Tukey 1.5 × IQR rule is non-parametric and robust to skew/heavy tails; using both reduces reliance on a single distributional assumption. Applying these criteria led to 69/167 exclusions (41%). Because repeated-measures ANOVA requires complete cases, we used listwise deletion for missing T2 (*n* = 1) or T3 (*n* = 52) data. Randomization was preserved, and included vs. excluded participants did not differ systematically on baseline sociodemographic or psychological variables. The final analytic sample was *N* = 98 (~30 per group).

Robustness and ITT analyses: (a) we re-estimated the RM-ANOVA models including the previously excluded cases; the overall pattern of results was unchanged, with some attenuation of effect sizes (Table 2); and (b) we fitted intention-to-treat linear mixed-effects models to all randomized participants (*N* = 167) using maximum-likelihood estimation and all available observations (T1–T3), with fixed effects of Group, Time, and Group × Time and participant-level random intercepts. This ITT approach avoids casewise deletion and yields consistent estimates under a missing-at-random assumption. The ITT results reproduced the primary patterns (see Results). To probe generalizability, we added Gender (female vs. male) and its interactions with Time and Group to the complete-case RM-ANOVA and the ITT mixed-effects models (fixed effects: Group, Time, Group × Time, Gender, Gender × Time, Gender × Group, Gender × Group × Time). These moderation tests were exploratory and underpowered given the small number of men (*n* = 13).

The initial data set comprised 167 individuals. After conducting a rigorous outlier analysis using both the *Z*-score method and the interquartile range (IQR) method, 69 participants were classified as outliers and subsequently excluded, reducing the final sample size to *N* = 98. Participants with missing data in T2 (*n* = 1) and T3 (*n* = 52) were excluded to meet the requirement for the *repeated-measures analysis of* var*iance* (RM-ANOVA). Sensitivity analyses comparing participants with complete data to those with missing data showed no significant differences in socio-demographic and baseline characteristics, suggesting that the exclusion did not introduce any systematic bias.

While the outlier exclusion followed strict methodological standards to ensure the validity of the findings, we acknowledge that the exclusion of 41% of participants is substantial. However, a sensitivity analysis was conducted, confirming that the main outcomes remained consistent even when these outliers were included, supporting the robustness of the findings. The 98 participants, enrolled in either a bachelor’s or master’s degree program at a distance learning university, were recruited anonymously via email in January 2023. The study team had no direct contact with participants during the registration process. Randomization ensured a balanced distribution across the experimental and control groups. The participants in experimental group A (EG_A, *N* = 30) had a mean age of 33.2 years (*SD* = 9.01), with 27 female and 3 male participants. Experimental group B (EG_B, *N* = 31) had a mean age of 37.8 years (*SD* = 10.44), with 25 female and 5 male participants. The control group (CG, *N* = 37) had a mean age of 36.2 years (*SD* = 8.46), comprising 33 female and 4 male participants. Approximately half of the participants were employed part-time, while a third were employed full-time. One-third of the participants reported having children. Three-quarters of the participants were enrolled in a bachelor’s program, and the remaining quarter in a master’s program. In terms of marital status, 29 participants were single, 28 were in a relationship, and 41 were married (Table 3). The efficacy of the randomization process was evaluated by conducting a comparative analysis of the groups on salient baseline sociodemographic and psychological variables, encompassing age, gender, employment status, parenthood, study programmeme, and marital status, in addition to the baseline psychological measures collected during the T1 assessment. A series of one-way ANOVAs and chi-square tests were conducted to examine group differences. The findings indicated that there were no statistically significant differences between the groups on these variables (*p* > 0.05), thereby confirming that the randomization process had effectively created comparable groups at the baseline stage.

**Table 3:** Demographic characteristics of participants by group.

Variable	Mind2Full (*n* = 30)	ComGrat (*n* = 31)	CG (*n* = 37)	Total (N = 98)
Gender				
Female	27	25	33	85
Male	3	6	4	13
Age				
Mean (SD)	33.2 (9.01)	37.8 (10.44)	36.2 (8.46)	35.8 (9.38)
Employment				
Full-time	11	11	14	36
Part-time	16	15	17	48
Unemployed	3	5	6	14
Children				
None	22	19	22	63
>1	8	12	15	35
Study program				
Bachelor	22	24	28	74
Master	8	7	9	24
Marital status				
Single	11	5	13	29
In a Relationship	7	10	11	28
Married	12	16	13	41

This table presents gender, age, employment status, parental status, study programme, and marital status for participants in the *Mind2Full*, *ComGrat*, and control groups. n = number of participants; SD = standard deviation; CG = Control Group.

The sample is considered representative of the broader population of distance learning students for several reasons. Firstly, research shows that a significant proportion of distance learners are concurrently employed, often balancing academic responsibilities with professional and personal obligations (Åkerfeldt et al., 2024), as reflected in the present sample, where 85% of participants were employed part-time or full-time. This finding is consistent with prior research that emphasizes the dual commitments of work and study as a hallmark of distance learning populations (Kahu et al., 2013; Stone, 2017). Secondly, the substantial initial sample size of 254 invited participants, along with a final sample of 98 participants, serves to enhance the representativeness of the study, thereby reducing selection bias and ensuring the reliability of the findings. The preponderance of female participants in the study, constituting approximately 87% of the sample, aligns with the findings of prior studies that attribute this imbalance to the increased engagement of women in online and distance education due to its inherent flexibility (Stone, 2017). This demographic composition reflects broader trends in distance learning and lends confidence to the generalizability of the findings to similar populations.

## 3 Results

The results are organized by outcome variable—mindfulness skills, self-compassion, and perceived stress—and are summarized in Table 4 (means and standard deviations by group and time point) and Table 5 (mixed factorial ANOVA results).

**Table 4:** Means and standard deviations for mindfulness, self-compassion, and perceived stress across all groups (*Mind2Full*, *ComGrat*, Control) at three time points (T1 = baseline, T2 = post-intervention, T3 = 3-month follow-up).

Outcome	Group	T1 Mean (SD)	T2 Mean (SD)	T3 Mean (SD)
Mindfulness (CHIME)	*Mind2Full*	3.21 (0.45)	3.80 (0.39)	3.56 (0.41)
	*ComGrat*	3.18 (0.42)	3.61 (0.37)	3.68 (0.35)
	Control	3.17 (0.43)	3.19 (0.41)	3.22 (0.44)
Self-compassion (SCS)	*Mind2Full*	2.84 (0.37)	3.19 (0.34)	3.03 (0.36)
	*ComGrat*	2.86 (0.39)	3.17 (0.36)	3.05 (0.34)
	Control	2.81 (0.40)	2.84 (0.38)	2.83 (0.39)
Perceived stress (PSQ)	*Mind2Full*	2.91 (0.41)	2.48 (0.38)	2.70 (0.40)
	*ComGrat*	2.94 (0.43)	2.56 (0.36)	2.57 (0.37)
	Control	2.88 (0.40)	2.86 (0.41)	2.83 (0.42)

**Table 5:** Summary of mixed factorial ANOVA results for each outcome variable, including main effects of time and group, as well as time × group interaction effects.

Outcome variable	Effect type	*F*-value	df	*p*-value	Partial η^2^
Mindfulness (CHIME)	Time	36.2	2, 190	<0.001	0.276
Mindfulness (CHIME)	Group	10.4	2, 95	<0.001	0.179
Mindfulness (CHIME)	Time × Group	10.7	4, 190	<0.001	0.184
Self-compassion (SCS)	Time	5.34	1.88, 178.6	0.006	0.053
Self-compassion (SCS)	Group	3.12	2, 95	0.049	0.062
Self-compassion (SCS)	Time × Group	2.58	3.76, 83.07	0.042	0.051
Perceived stress (PSQ)	Time	27.8	2, 190	<0.001	0.226
Perceived stress (PSQ)	Group	6.12	2, 95	0.003	0.114
Perceived stress (PSQ)	Time × Group	6.45	4, 190	<0.001	0.119

Values reported include *F*-values, *degrees of freedom* (df), *p*-values, and *partial eta squared* (η^2^).

Control group T3 values reflect immediate post-intervention scores following delayed access to the intervention and are not comparable to follow-up scores from the intervention groups. Accordingly, all T3 “maintenance” statements refer to within-group change relative to baseline, and between-group comparisons at T3 are restricted to the two active arms; the control group is not included in T3 between-group tests.

### 3.1 Mindfulness skills

Changes in mindfulness skills, as measured by the CHIME scale, were examined across the *Mind2Full* (experimental group A), *ComGrat* (experimental group B), and control groups over three time points (T1, T2, and T3). Mauchly’s test confirmed that the assumption of sphericity was met, χ^2^(2) = 0.993, *p* = 0.724; therefore, no correction to degrees of freedom was required. The assumptions of normality, homogeneity of variances, and independence of observations were satisfied. A 3 (Group) × 3 (Time) repeated measures ANOVA revealed a significant interaction effect between time and group, *F*(4, 190) = 10.7, *p* < 0.001, η^2^ₚ = 0.184, indicating a large effect size and suggesting that changes in mindfulness skills over time differed by group.

From T1 to T2, both intervention groups demonstrated statistically significant improvements in mindfulness scores. Participants in the *Mind2Full* group showed a mean increase of 0.594 points on the CHIME scale, *t*(95) = 7.587, *p* < 0.001, 95% CI [0.533, 0.654], while participants in the *ComGrat* group improved by 0.428 points, *t*(95) = 5.566, *p* < 0.001, 95% CI [0.385, 0.472]. In contrast, the control group did not exhibit a significant change during this period. *Post hoc* pairwise comparisons at T2 (Tukey) indicated that CHIME scores were higher in *Mind2Full* than control, *t*(95) = 5.592, *p* < 0.001, and higher in *ComGrat* than control, *t*(95) = 3.131, *p* = 0.002. The *Mind2Full* vs. *ComGrat* contrast at T2 was not significant (*p* > 0.05).

**Figure 1:**
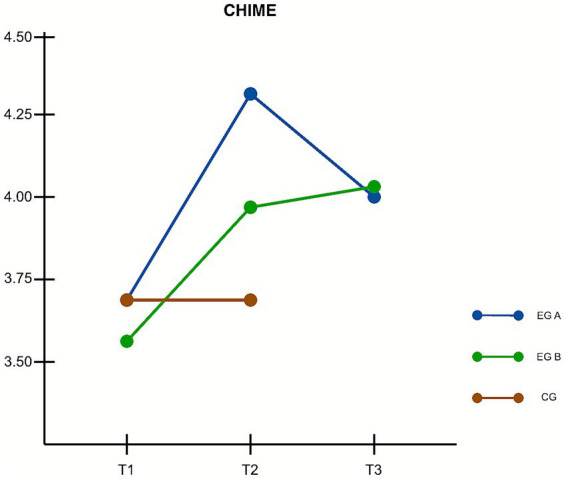
Changes in mindfulness skills (CHIME scores) over time. Estimated marginal means are shown for three time points: baseline (T1), post-intervention (T2), and three-month follow-up (T3). EG A = *Mind2Full*, EG B = *ComGrat*, CG = Control Group. Both intervention groups showed increases in mindfulness from T1 to T2. At T3, the *ComGrat* group maintained higher scores, while *Mind2Full* scores slightly declined.

Long-term effects from T1 to T3 were also observed. The *Mind2Full* group maintained a statistically significant gain, with a mean increase of 0.348 points, *t*(95) = 4.344, *p* = 0.001, 95% CI [0.312, 0.383]. The *ComGrat* group exhibited an even larger improvement of 0.497 points, *t*(95) = 6.313, *p* < 0.001, 95% CI [0.446, 0.548]. Although both interventions led to sustained increases in mindfulness scores, the *ComGrat* group achieved a more pronounced long-term effect than *Mind2Full*. These findings support hypothesis H1a and partially support H1b, demonstrating significant short-term gains in mindfulness skills in both intervention groups. Hypothesis H1c was partially supported, as *ComGrat* showed greater long-term improvement than *Mind2Full*, contrary to the initial expectation. Figure 1 displays the estimated marginal means of CHIME scores across the three time points.

### 3.2 Results—self-compassion

Changes in self-compassion were assessed using the SCS scale across the *Mind2Full*, *ComGrat*, and control groups at baseline (T1), immediately after the intervention (T2), and at three-month follow-up (T3). Mauchly’s test indicated that the assumption of sphericity was violated, χ^2^(2) = 0.936, *p* = 0.045; therefore, Greenhouse–Geisser corrections were applied (*ε* = 0.940). A repeated measures ANOVA revealed a statistically significant interaction between time and group, *F*(3.76, 83.07) = 2.58, *p* = 0.042, η^2^ₚ = 0.051, indicating a small effect size and suggesting differential changes in self-compassion over time across the three groups.

Between T1 and T2, both intervention groups showed significant improvements in self-compassion scores. *Mind2Full*: mean increase = 0.351, *t*(95) = 4.482, *p* < 0.001, 95% CI [0.316, 0.386]; *ComGrat*: mean increase = 0.308, *t*(95) = 4.183, *p* = 0.002, 95% CI [0.277, 0.338]. No significant change was observed in the control group during this period. However, *post hoc* comparisons revealed no statistically significant differences between either intervention group and the control group at T2. This finding indicates that although participants in both interventions improved significantly relative to their own baselines, these gains were not large enough to produce significant between-group effects at this time point. As such, hypothesis H2a was not supported.

Regarding T1 to T2 comparisons between interventions, the results did not show that *ComGrat* produced greater improvements than *Mind2Full*. Although the *ComGrat* group showed a slightly smaller mean gain, the difference between groups was not statistically significant. This partially supports hypothesis H2b, as both interventions enhanced self-compassion but neither demonstrated clear superiority immediately following the intervention.

**Figure 2:**
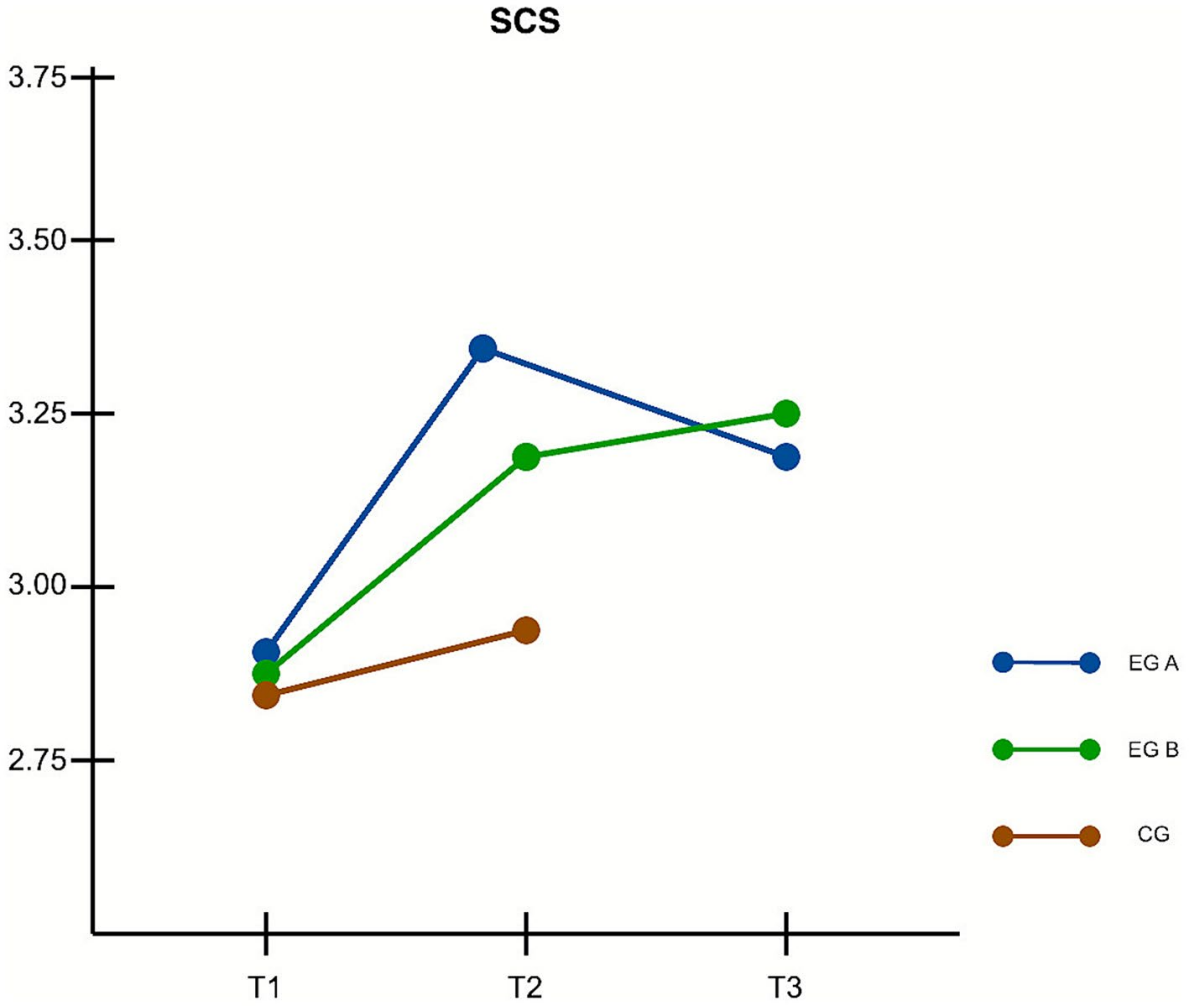
Changes in self-compassion skills (SCS scores) over time. Estimated marginal means are shown for baseline (T1), post-intervention (T2), and three-month follow-up (T3). EG A = *Mind2Full*, EG B = *ComGrat*, CG = Control Group. Both intervention groups showed significant increases in self-compassion from T1 to T2. At T3, the *ComGrat* group retained more of its initial improvement, whereas the *Mind2Full* group showed a decline, suggesting greater long-term effects for *ComGrat*.

In terms of long-term effects from T1 to T3, both intervention groups showed declines in self-compassion relative to their post-intervention levels. Compared to baseline, the *Mind2Full* group exhibited a net reduction, while the *ComGrat* group maintained a modest gain of approximately 0.19 points. Although this does not constitute a statistically significant improvement over baseline, it suggests that participants in the *ComGrat* group retained more of their initial benefit than those in the *Mind2Full* group. As such, hypothesis H2c is only partially supported. Figure 2 presents the estimated marginal means of SCS scores across the three time points.

### 3.3 Results—perceived stress

Perceived stress was assessed using the PSQ scale across the *Mind2Full*, *ComGrat*, and control groups at baseline (T1), immediately after the intervention (T2), and at three-month follow-up (T3) for the intervention groups. The control group completed a third assessment (T3), but this measurement reflects immediate post-intervention effects following delayed access to the program, rather than a true follow-up. Mauchly’s test indicated that the assumption of sphericity was met, so no correction to the degrees of freedom was required. A 3 (Group) × 3 (Time) repeated measures ANOVA revealed a significant interaction effect between time and group, *F*(4, 190) = 6.45, *p* < 0.001, η^2^ₚ = 0.119, indicating a moderate effect size. From T1 to T2, both intervention groups showed meaningful reductions in perceived stress, consistent with the observed PSQ changes. Participants in the *Mind2Full* group exhibited a mean increase of 0.43 points, while the *ComGrat* group improved by 0.38 points. These results confirm that both interventions were effective in reducing perceived stress in the short term, supporting hypothesis H3a.

At the three-month follow-up (T3), improvements were partly sustained. The *Mind2Full* group maintained a smaller, yet still positive, gain of 0.21 points from baseline, while the *ComGrat* group retained most of its initial benefit, with a gain of 0.37 points from T1. These findings suggest modest but persistent reductions in perceived stress following both interventions. The more stable outcome observed in the *ComGrat* group lends partial support to hypothesis H3c, which anticipated stronger long-term effects for that intervention. In contrast, hypothesis H3b, which predicted greater short-term reductions in perceived stress for *Mind2Full* compared to *ComGrat*, was not supported, as the two groups showed comparable effects.

**Figure 3:**
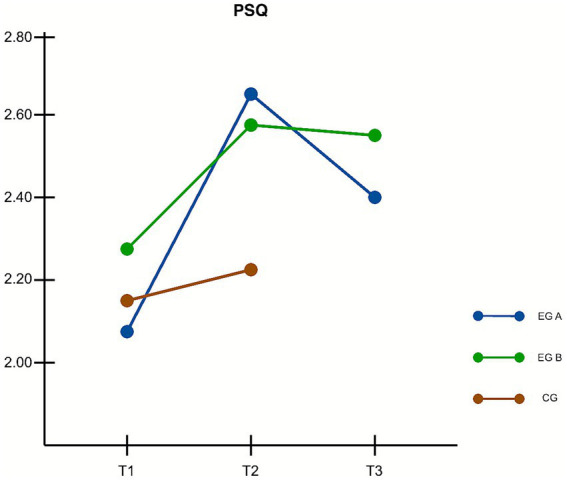
Changes in perceived stress (PSQ scores) over time. Estimated marginal means are shown for baseline (T1), post-intervention (T2), and three-month follow-up (T3). EG A = *Mind2Full*, EG B = *ComGrat*, CG = Control Group. Both intervention groups showed significant short-term reductions in perceived stress from T1 to T2. At T3, the *ComGrat* group maintained most of its gains, while the *Mind2Full* group showed a partial decline, indicating more stable long-term benefits for *ComGrat*.

Overall, both interventions were successful in reducing perceived stress, with *ComGrat* showing a slightly more stable long-term profile. Figure 3 displays the estimated marginal means of PSQ scores across the three time points.

The study found significant improvements in perceived stress across both experimental groups (*Mind2Full* and *ComGrat*) immediately after the interventions (T2) and 3 months later (T3). An analysis of the four PSQ subscales—Worries, Tension, Joy, and Demands—showed significant improvements over time in all subscales: Worries, *F*(2, 96) = 13.41, *p* < 0.001, η^2^ₚ = 0.125; Tension, *F*(2, 96) = 24.80, *p* < 0.001, η^2^ₚ = 0.205; Joy, *F*(2, 96) = 8.44, *p* < 0.001, η^2^ₚ = 0.081; and Demands, *F*(2, 96) = 14.81, *p* < 0.001, η^2^ₚ = 0.134. Short-term (T1 → T2) improvements were: Worries [MD = −0.3049, *t*(95) = −4.214, *p* < 0.001], Tension [MD = −0.5672, *t*(95) = −8.578, *p* < 0.001], Joy [MD = −0.2721, *t*(95) = −4.260, *p* < 0.001], and Demands [MD = −0.4787, *t*(95) = −6.301, *p* < 0.001]. Long-term (T1 → T3) effects were observed only in *ComGrat*: Worries [MD = −0.394, *t*(95) = −3.351, *p* = 0.030], Tension [MD = −0.413, *t*(95) = −3.861, *p* = 0.006], and Demands [MD = −0.3135, *t*(95) = −3.275, *p* = 0.018]; Joy did not maintain significance at follow-up.

Regarding H3d, these results support the hypothesis that the *ComGrat* intervention would lead to more sustained benefits, particularly in reducing Worries, Tension, and Demands. The anticipated reduction in the effectiveness over time was also observed, especially in the Joy subscale, which did not show long-term significance. The findings suggest that, while both interventions were effective in the short term, the *ComGrat* intervention had a more durable impact on reducing perceived stress in the specific subscales of Worries, Tension, and Demands. The decline in positive outcomes between T2 and T3 can be attributed to the three-month interval, during which the immediate benefits of the mindfulness interventions naturally diminished. This pattern is common in mindfulness-based interventions, as improvements in stress reduction tend to decrease unless the practices are regularly maintained. This trend aligns with existing research indicating that ongoing mindfulness practice is necessary to sustain long-term benefits (Baer, 2003).

To implement an intention-to-treat (ITT) analysis, we fitted linear mixed-effects models to all randomized participants (*N* = 167) using all available observations (T1–T3) with maximum-likelihood estimation, fixed effects of Group, Time, and Group × Time, and participant-level random intercepts. Results replicated the primary pattern: clear Group × Time interactions for mindfulness (CHIME) and perceived stress (PSQ), and small, directionally consistent effects for self-compassion (SCS). Effect magnitudes were modestly attenuated relative to the complete-case ANOVA; substantive conclusions were unchanged.

In exploratory gender moderation analyses, neither the Gender × Group × Time interaction nor any lower-order interactions involving Gender reached statistical significance at *α* = 0.05 in either the complete-case RM-ANOVA or the ITT mixed-effects models. Given the small number of male participants (*n* = 13), these null findings should be interpreted cautiously and considered underpowered.

## 4 Discussion

The present study explores the prevalence of stress among students, particularly in the context of the transition to distance learning and the escalating digitalization that has accompanied it. This shift has had a significant impact on mental health and academic performance, constituting a substantial challenge for students. The present study evaluated two self-directed web-based mindfulness interventions. The *Mind2Full* programme encompasses a comprehensive range of mindfulness skills, whereas the *ComGrat* programmeme focuses on self-compassion and gratitude. The objective of the study was to ascertain whether a focused intervention would yield more long-term benefits in accordance with the tenets of cognitive load theory. We interpret the findings in relation to our preregistered hypotheses. For clarity, we use ‘maintenance’ to denote within-group retention of gains from T1 to T3, and ‘durability’ to denote comparative patterns between the two active arms at T3; we do not claim long-term differences versus control because the wait-list received the intervention after T2.

Hypothesis appraisal (noting design constraints at T3): Because the wait-list received the intervention after T2, there is no untreated control at T3; thus all T3 “maintenance” refers to within-arm change, and T3 between-group contrasts are restricted to the two active arms.

H1 (Mindfulness). H1a (both interventions > control at T2) was supported; the time × group interaction for CHIME was significant and both intervention groups showed short-term gains. H1b (*Mind2Full* > *ComGrat* at T2) received partial support: both groups improved similarly at T2, while *ComGrat* showed the more durable profile at follow-up. H1c (both groups maintain gains at T3, stronger for *Mind2Full*) was partially supported as within-arm maintenance: both groups retained improvements, but maintenance was relatively stronger for *ComGrat*.

H2 (Self-compassion). H2a (both interventions > control at T2) was not supported in between-group contrasts despite clear within-arm gains. H2b (*ComGrat* > *Mind2Full* at T2) was not supported; immediate post-intervention improvements were comparable. H2c (*ComGrat* > *Mind2Full* at T3) received partial support: *ComGrat* retained more of its initial improvement within-arm, although T3 between-arm contrasts did not consistently reach significance.

H3 (Perceived stress). H3a (both interventions reduce stress at T2 vs. control) was supported. H3b (*Mind2Full* > *ComGrat* at T2) was not supported; short-term effects were similar. H3c (*ComGrat* > *Mind2Full* at T3) was partially supported as within-arm durability: *ComGrat* exhibited the more stable long-term profile, with modest T3 between-arm differences.

Effect size interpretation and comparison: Interpreting the partial eta-squared (η^2^p) values using Cohen’s *f* (conversion *f* = √[η^2^p/(1 − η^2^p)]), the time × group interaction effects were large for mindfulness (CHIME: η^2^p = 0.184 → *f* ≈ 0.48), between medium and large for perceived stress (PSQ: η^2^p = 0.119 → *f* ≈ 0.37), and small-to-medium for self-compassion (SCS: η^2^p = 0.051 → *f* ≈ 0.23). Main effects of time were large for CHIME (η^2^p = 0.276 → *f* ≈ 0.62) and PSQ (η^2^p = 0.226 → *f* ≈ 0.54), and small-to-medium for SCS (η^2^p = 0.053 → *f* ≈ 0.24). Taken together, these magnitudes are broadly consistent with the small-to-moderate effects commonly reported in student samples for digital mindfulness, self-compassion, and gratitude-based interventions, with our mindfulness and stress outcomes trending toward the upper end of that range. The comparatively smaller interaction for self-compassion aligns with the observed attenuation from T2 to T3 and the absence of consistent between-group differences at follow-up.

Both interventions demonstrated significant within-group improvements in perceived stress and self-compassion over time. Because the wait-list received the program after T2, no untreated control exists at T3; thus all “maintenance” at T3 is interpreted within each intervention arm. On this basis, *ComGrat* showed a more stable within-group trajectory than *Mind2Full*. Between-group contrasts are therefore limited to T2: both interventions outperformed control on mindfulness and perceived stress, whereas self-compassion differences versus control did not consistently reach significance. In addition, between-group comparisons did not consistently reveal statistically significant differences between *Mind2Full* and control. These patterns underscore the importance of distinguishing within-group change from between-group effects when interpreting intervention efficacy. The observed sustained pattern in the *ComGrat* arm supports the potential value of targeted interventions focused on self-compassion and gratitude; however, direct long-term (T3) comparisons with the control group are not available under this design. Control group T3 data reflect immediate post-intervention effects following delayed participation and were used to assess short-term benefits in a delayed-exposure cohort; this design necessarily precluded long-term comparisons between intervention and control groups.

This interpretation aligns with the tenets of CLT, which emphasize the reduction of extraneous cognitive load to enable learners to focus on core concepts. The study found that both interventions led to significant within-group reductions in stress levels over time. However, only the *ComGrat* program displayed more pronounced within-group maintenance to the three-month follow-up and a more stable trajectory than *Mind2Full*. Because the control group began treatment after T2, long-term comparative efficacy versus an untreated control cannot be established. The more focused approach of *ComGrat* likely minimized extraneous cognitive load, allowing for deeper cognitive processing and more robust learning outcomes over time. The long-term effects observed in the *ComGrat* group are consistent with prior research demonstrating that self-compassion interventions, such as Mindful Self-Compassion programs, can significantly enhance emotional resilience and coping mechanisms (Ferrari et al., 2019; Neff and Germer, 2021). However, this study builds on earlier findings by directly comparing a targeted intervention to a broader mindfulness program. The results reveal that the focused approach of *ComGrat* not only reduces stress but also leads to greater long-term benefits. This finding lends support to the tenets of Cognitive Load Theory (Sweller, 2010), which posits that the reduction of extraneous cognitive load enables learners to engage more profoundly with fundamental concepts. While broader interventions, such as Mindfulness-Based Stress Reduction (MBSR), have been found to be effective, they may introduce an excessive number of elements simultaneously, thereby potentially diminishing their long-term impact. By contrast, the concentrated approach of the *ComGrat* programmeme is likely to have contributed to its effectiveness by reducing cognitive load, thereby allowing for deeper engagement with the material. Conversely, the *Mind2Full* intervention, despite its initial efficacy, might have resulted in cognitive overload, thereby impeding its long-term impact.

Nevertheless, the absence of any discernible enhancement in specific aspects of mindfulness within the *Mind2Full* group necessitates further scrutiny. One potential explanation for this phenomenon is that the diversity of mindfulness exercises employed in *Mind2Full* may have hindered participants’ ability to internalize any one skill profoundly. Research suggests that the introduction of too many new techniques in a short time can increase cognitive demand, reducing retention and long-term engagement (Sweller, 2010). It is possible that participants experienced difficulties in sustaining consistent practice across all mindfulness dimensions, particularly in the absence of personalized feedback or follow-up support. Alternatively, the measurement tools employed in this study, although validated, may have lacked the sensitivity to detect subtle yet significant changes in specific mindfulness dimensions over time. This finding is consistent with prior research demonstrating that certain mindfulness dimensions, such as decentering and non-judgmental awareness, necessitate extended practice to manifest sustained improvements (Shapiro et al., 2005). It is recommended that subsequent studies extend the duration of the intervention or incorporate additional assessments to capture subtle or delayed changes. Finally, individual differences among participants, such as baseline mindfulness levels or varying levels of engagement with specific exercises, may have influenced the results. These factors could provide a rationale for why certain dimensions of mindfulness exhibited less consistent improvement in the *Mind2Full* group, despite the program’s comprehensive design. In subsequent research, the exploration of moderators such as personality traits or baseline stress levels could offer a more profound understanding of these findings.

The focus of *ComGrat*, which was on self-compassion and gratitude, is likely to have enabled participants to engage more thoroughly with these concepts. This may have resulted in greater emotional regulation and stress reduction. Self-compassion fosters emotional resilience by encouraging individuals to respond to personal setbacks with understanding and kindness rather than self-criticism, a skill that is particularly beneficial for distance learners managing multiple responsibilities (Neff and Germer, 2021). Gratitude, in this sense, serves to promote a positive shift in attention, focusing on affirming and appreciating positive aspects of life rather than stressors (Emmons and McCullough, 2003). The observed disparities in the long-term improvements in self-compassion and perceived stress between the *ComGrat* participants and the broader *Mind2Full* group may be attributable to these psychological shifts. Furthermore, targeted interventions such as *ComGrat* have the potential to yield more sustained benefits by virtue of the fact that they reduce the cognitive and emotional effort required to process multiple concepts simultaneously, as posited by *CLT*. This narrower focus allows participants to internalize specific skills more effectively and to practice them consistently over time, thereby fostering stronger neural consolidation and habit formation (Sweller, 2010; Karpicke and Roediger, 2008). This interpretation is further substantiated by prior research, which demonstrates that focused interventions frequently result in more profound emotional engagement and enhanced long-term retention in comparison to broader approaches (Cregg and Cheavens, 2021; Baer, 2003). While the targeted nature of *ComGrat* appears to confer advantages in long-term retention and emotional resilience, it may also limit exposure to the breadth of mindfulness practices that more comprehensive programs like *Mind2Full* offer. This narrower focus might not address all facets of stress-related challenges or individual differences in preference and responsiveness to specific techniques. Consequently, some participants may benefit more from a broader repertoire of skills, particularly if their stressors are varied and multifaceted. Future research could explore whether combining focused and comprehensive approaches might provide more holistic benefits for diverse learner populations.

Furthermore, gratitude and self-compassion have been demonstrated to facilitate positive feedback loops that reinforce emotional wellbeing (Cregg and Cheavens, 2021). For instance, expressing gratitude has been associated with enhanced social connection and positive affect, which, in turn, have been shown to reduce stress levels (Cregg and Cheavens, 2021). In a similar vein, the practice of self-compassion has been demonstrated to curtail rumination and self-criticism, thereby fostering emotional resilience (Neff K., 2003; Neff K. D., 2003). It is plausible that these mechanisms contributed to the sustained benefits observed in the *ComGrat* group, particularly in reducing perceived stress and enhancing self-compassion over time. In addition to CLT, Spaced Repetition Theory (SRT) provides further explanatory value. SRT posits that learning is enhanced when key material is encountered multiple times across intervals. The *ComGrat* intervention included repeated daily exposure to the same core concepts—self-compassion and gratitude—over the course of 4 weeks, which likely reinforced these skills through spaced repetition. This mechanism could have amplified consolidation and memory retention, particularly in emotionally salient contexts like stress management. Conversely, the *Mind2Full* program covered a broader range of techniques, which were less likely to benefit from this kind of structured repetition. The combined insights from CLT and SRT offer a robust theoretical rationale for *ComGrat*’s superior long-term outcomes.

The interaction between Cognitive Load Theory (*CLT*) and Spaced Repetition Theory (*SRT*) provides a more comprehensive explanation for the superior long-term outcomes of the *ComGrat* programmeme. As argued above, *CLT* emphasizes the importance of minimizing extraneous cognitive load to optimize learning outcomes (Sweller, 2010). Concurrently, Spaced Repetition Theory (*SRT*) emphasizes the pivotal function of repeated exposure to pivotal material in fostering long-term retention (Cepeda et al., 2006). The *ComGrat* programmeme’s daily repetition of self-compassion and gratitude exercises aligns directly with *SRT* principles, ensuring that participants not only engage with these practices but also revisit and internalize them consistently over time. The simplicity and focus of *ComGrat* likely facilitated deeper consolidation of emotional skills, as repeated practice strengthens neural pathways associated with these concepts (Gál et al., 2021). In contrast, the exercises of the *Mind2Full* program may not have provided participants with sufficient repetition to consolidate the extensive array of mindfulness skills presented. The integration of these frameworks elucidates the underlying reasons for *ComGrat*’s superior long-term outcomes. The reduction in cognitive overload (*CLT*) facilitated focused engagement with the intervention’s fundamental components, while the systematic repetition of practices (*SRT*) ensured the retention and utilization of these emotional skills over time. The integration of these theoretical frameworks into the *ComGrat* program exemplifies the potential for a synergistic integration of psychological and educational principles to enhance the efficacy of mindfulness interventions.

The findings outlined here demonstrate practical implications for the design of mental health interventions, particularly for distance learners. Integrating self-compassion and gratitude within mindfulness programs represents a targeted strategy that educational institutions and mental health professionals can implement to support students facing social isolation and multiple responsibilities. The superior long-term outcomes of *ComGrat* suggest that such focused interventions could be embedded in university wellness programs, online learning platforms, or student counselling services. For example, institutions could offer structured, asynchronous interventions via email or learning management systems, allowing students to engage flexibly at their own ace. Additionally, brief self-compassion and gratitude exercises can be integrated into existing curricula—such as stress-management courses or digital study guides—to enhance accessibility and effectiveness. Future research should examine the feasibility and scalability of these approaches across educational settings to maximize their impact on student wellbeing. While these delivery channels are promising, the heavily female and single-institution sample constrains generalizability—particularly to male students and on-campus populations. Scaling studies should therefore use multi-site recruitment, balanced gender quotas or targets, and preplanned, adequately powered moderation tests (e.g., Gender × Group × Time) to evaluate heterogeneity of effects.

The findings of this study add to the growing body of research highlighting the unique stressors faced by distance learners, such as social isolation, role conflict, and the lack of immediate support networks (Lawrence et al., 2019). These stressors are further compounded by the self-directed nature of online education, which requires heightened self-discipline and time management skills, often leading to increased levels of stress and burnout (Kahu et al., 2013). Mindfulness-based interventions, notably targeted approaches such as *ComGrat*, have been shown to address these challenges by cultivating emotional resilience and equipping students with the necessary skills to manage their stress independently. The findings emphasize the necessity for tailored interventions to meet the specific needs of distance learners. Web-based programs such as *Mind2Full* and *ComGrat*, which are characterized by their flexibility and accessibility, are especially well-suited to distance learners. In contrast to conventional face-to-face interventions, which often necessitate rigid scheduling and physical presence, web-based programs offer learners the autonomy to progress at their own pace and seamlessly integrate mindfulness practices into their daily routines. This finding is consistent with the conclusions other studies (Xiang et al., 2020; Flett et al., 2019), which emphasize the efficacy of digital interventions in reaching underserved populations.

Furthermore, the emphasis on self-compassion and gratitude in *ComGrat* directly addresses some of the psychological challenges unique to distance learners. Self-compassion has been shown to mitigate the negative effects of self-criticism and isolation (Cregg and Cheavens, 2021), while gratitude has been demonstrated to promote a positive outlook, which can counter feelings of disconnection and overwhelm (Wood et al., 2010). The provision of these skills to students through interventions such as *ComGrat* serves to reduce stress levels and enhance overall wellbeing, thereby fostering a sense of connection and self-efficacy, even in the absence of traditional support networks. These findings have practical implications for educational institutions aiming to support distance learners. In order to support distance learners, universities could integrate targeted interventions such as *ComGrat* into their onboarding or wellness programs. For instance, *ComGrat* could be implemented as a supplemental resource for first-year distance learners, helping them to build the emotional and cognitive tools necessary to navigate the challenges of online education. Furthermore, the combination of these interventions with broader institutional strategies, such as peer mentoring programs or virtual community-building initiatives, has the potential to further enhance their effectiveness by addressing both individual and systemic stressors faced by distance learners. Such programs could be supplemented with psychoeducational modules that elucidate the benefits of mindfulness, self-compassion, and gratitude, thereby ensuring a theoretical foundation to complement practice.

Another potential implementation could involve incorporating *ComGrat* into existing wellness programs or student support services, where students access these interventions via university learning management systems (LMS) or standalone mobile applications. These platforms offer flexibility by enabling students to engage in 15–20 min daily practices at their convenience while offering features such as reminders or progress tracking to maintain engagement. This flexibility is of particular benefit to distance learners, who often have irregular schedules and competing responsibilities. In addition, web-based platforms have demonstrated significant advantages in terms of cognitive therapy interventions (Xiang et al., 2020), whilst also offering a high level of accessibility and flexibility. The flexibility is particularly beneficial for distance learners, enabling them to incorporate mindfulness practices into their daily routines and revisit materials as needed (Dear et al., 2019; Yarnell et al., 2019). These findings highlight the value of tailoring digital mindfulness interventions to the cognitive and emotional profiles of specific learner groups, and suggest that incorporating principles from Cognitive Load Theory (CLT) and Spaced Repetition Theory (SRT) can guide the design of more effective, scalable programs. Future studies should continue to explore the potential of integrating theories such as *CLT* and *SRT* to optimize the design and implementation of mindfulness interventions.

### 4.1 Limitations and future directions

Notwithstanding the encouraging outcomes, the study is subject to several constraints.

First, the overrepresentation of female participants (~87%) from a single distance-learning institution raises questions about generalizability—especially to male students and on-campus populations. This gender imbalance is consistent with prior research showing that women are more likely to engage in mindfulness and self-compassion interventions (Yarnell et al., 2019) and may have influenced overall effects. To probe this, we ran exploratory moderation models adding Gender and its interactions with Time and Group in both the complete-case RM-ANOVA and the ITT mixed-effects analyses; no interaction terms reached significance at *α* = 0.05, but these tests were underpowered given the small number of men (*n* = 13). Future work should recruit balanced, multi-site samples and pre-register adequately powered moderation tests to examine potential gender-based variation in outcomes.

Second, outliers were identified separately for each outcome (CHIME, PSQ, SCS) at T2 and T3 using pre-specified |*Z*| ≥ 3 and 1.5 × IQR rules, applied uniformly before any group comparisons. This led to the exclusion of 69/167 participants (41%). Because RM-ANOVA requires complete cases, we also used listwise deletion for missing T2 (*n* = 1) and T3 (*n* = 52) data. Randomization was preserved, and included vs. excluded participants did not differ systematically on baseline socio-demographic or psychological measures. To gauge impact, we re-estimated all models including the excluded cases and, in a separate robustness check, fit linear mixed-effects models using all available observations; across checks, the direction and pattern of results were unchanged, with some attenuation of effect sizes (see Table 2). Even so, the extent of case loss may introduce selection bias, reduce precision, and limit external validity. Although the ITT mixed-effects analysis using all randomized participants reproduced the main patterns, residual bias cannot be ruled out if the missingness mechanism deviated from missing-at-random (MAR). Future preregistered studies should prioritize mixed-effects/robust estimators with maximum-likelihood handling of missingness to minimize case loss and improve generalizability.

Third, because participants self-assessed their own physical and mental fitness at enrollment and no health-related exclusion criteria were applied, unmeasured medical or psychological conditions may have influenced stress or mindfulness outcomes. While this decision was intended to preserve ecological validity and inclusiveness in a preventive, low-risk intervention, future studies should consider stratifying or controlling for health status to examine potential moderating effects.

Fourth, this study used repeated measures ANOVA, selected for its suitability with balanced data and alignment with prior research (Rana et al., 2022; Bock et al., 2024; Bock et al., 2025). Greenhouse–Geisser corrections were applied where sphericity was violated. While appropriate for the present design, linear mixed models (LMM) would offer greater flexibility to handle individual variability, unequal spacing, and missing-at-random data; future work could adopt LMM to model intra-individual trajectories (including random effects and unknown sources of secondary variance), thereby extending generalizability and clarifying differential intervention effects. We also note that skewness and kurtosis were not tabulated for the outcome distributions; although residual diagnostics did not indicate material departures from normality, future studies may report these indices (e.g., in Supplementary materials) for completeness.

Fifth, the final group sizes (~30 per group) may have reduced the statistical power to detect between-group differences, particularly for small effect sizes. This may partly explain why some comparisons did not reach statistical significance despite observed within-group improvements. In particular, while within-group improvements in perceived stress and self-compassion were observed, the absence of statistically significant between-group differences limits the extent to which these effects can be causally attributed to the interventions.

Sixth, the utilization of self-reported data carries the potential for response bias, including social desirability and recall errors. Participants may have overestimated their adherence to the intervention or reported greater improvements in stress and mindfulness skills due to a desire to provide favorable feedback. The incorporation of objective measures, such as physiological stress markers (e.g., cortisol levels or heart rate variability), and the utilization of digital engagement metrics (e.g., time spent on intervention activities) to track adherence would serve to triangulate self-reported data and provide a more robust assessment of intervention effectiveness. All outcome measures were analyzed using total scores, calculated as the average of relevant items (with reverse coding applied where appropriate). Although validated subscales exist, these were not included in the analysis, limiting the granularity of interpretation.

Seventh, the generalizability of these findings to more diverse populations of distance learners also warrants further exploration. Cultural or contextual factors, such as differing attitudes toward self-compassion and gratitude or variations in educational systems, may influence the effectiveness of these interventions. It is therefore recommended that future research evaluates the interventions in culturally diverse settings in order to determine whether their benefits extend across populations with differing values, norms, and stressors.

Eighth, although the three-month follow-up period is longer than in many similar studies, it may not have fully captured the long-term sustainability of the interventions’ benefits, particularly for *ComGrat*. A follow-up period of 6 or 12 months would facilitate a more comprehensive understanding of the longevity of the benefits. Furthermore, the efficacy of these interventions in more diverse populations, including individuals from different age groups, cultural backgrounds, and educational settings, should be investigated in future research.

Ninth, external validity and intervention fidelity warrant caution. The targeted web-based program was tested within a single institution and delivered via daily emails; we did not systematically monitor participants’ use of external mindfulness or self-help resources (e.g., apps, courses) during the study. As such, contamination cannot be ruled out, and the magnitude of effects may differ in other delivery channels or populations. Future trials should incorporate fidelity and contamination assessments (e.g., brief app-usage surveys, activity logs), pre-registered protocols, and multi-site samples to evaluate generalizability. In addition, while multiplicity was controlled through a hierarchical sequence (PSQ → CHIME → SCS) and Tukey-adjusted post-hoc tests within endpoints, exploratory analyses were not family-wise error controlled; these findings should therefore be interpreted cautiously, with emphasis on effect sizes and 95% CIs.

Finally, we did not include direct process measures of the proposed mechanisms (e.g., perceived cognitive load or spacing adherence). Consequently, any mechanism-of-action inferences remain provisional. Future work could incorporate validated cognitive-load indices and dose–response summaries of repetition to test these pathways more rigorously.

## 5 Conclusion

This study shows that both comprehensive (Mind2Full) and targeted (ComGrat) self-directed online mindfulness interventions reduced perceived stress and improved mindfulness in distance learners, with robust post-intervention gains. Because the wait-list received the program after T2, no untreated control exists at T3; therefore, long-term “maintenance” is interpreted within arms. On this basis, ComGrat exhibited a more durable within-arm profile at follow-up for perceived stress (and a more stable trajectory for mindfulness). Self-compassion gains attenuated by T3 and did not consistently differ between groups. These patterns, replicated in ITT mixed-effects models, support the promise of focused, theory-informed interventions that minimize cognitive load and leverage repetition, while highlighting the need for larger, multi-site trials with balanced gender representation to assess scalability and heterogeneity of effects.

## Data Availability

The raw data supporting the conclusions of this article will be made available by the authors, without undue reservation.

## Footnotes

1
Random.org
