# Supplementary material for: Targeted mindfulness and self-compassion improve long-term stress reduction in distance learning students: a randomized trial
Source: Front Psychol. 2026 Jan 14;16:1678094. doi: 10.3389/fpsyg.2025.1678094 (PMC12847346; doi:10.3389/fpsyg.2025.1678094)
Supplement: Supplementary file 1 [file Data_Sheet_1.pdf]

Examples of E-Mails from the ComGrat Course

Introduction Mail (German Version)

Willkommen zum vierwöchigen Kurs

Willkommen zum vierwöchigen Achtsamkeitskurs "Stressfrei durch das Studium". Achtsamkeit hat viele Facetten und in diesem Kurs werden wir uns hauptsächlich mit dem Teil: emotionale Kompetenzen, Empathie, Mitgefühl und Dankbarkeit beschäftigen.

Morgen kann es auch schon losgehen!

Ein paar Tipps für die Durchführung gebe ich dir vorab in diesem Video.

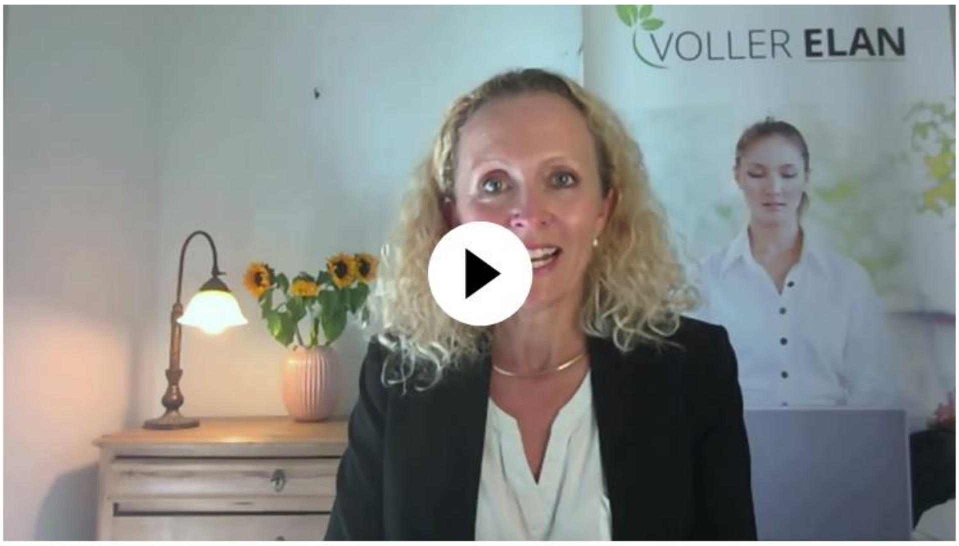

Wenn du wissen möchtest wie Empathie, Mitgefühl und Dankbarkeit mit den emotionalen Kompetenzen und der emotionale Intelligenz zusammen hängen, kannst du dir gerne vorher dieses Video anschauen. Hier erkläre ich ganz kurz, worum es geht.

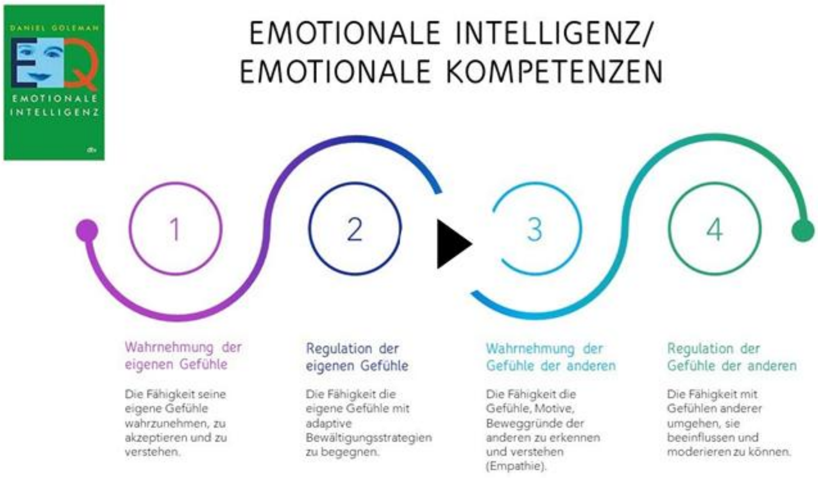

MORGEN GEHT ES LOS!

Schon morgen früh bekommst du die erste E-Mail des Kurses. In jeder E-Mail erhältst du ein Video mit einem kleinen Impuls für den Tag und ein Video mit einer Meditation. Abends bekommst du noch eine E-Mail mit einer Erinnerung an das Ausfüllen des Dankbarkeitstagebuches.

WICHTIGE HINWEISE VOR DEM KURS:

Das tägliche Programm dauert nicht mehr als 15-20 Minuten. Das ist nicht viel, aber du wirst feststellen, dass wahrscheinlich genau hierin die größte Herausforderung für dich bestehen wird, diese Zeit freizuräumen.

Also, tue dir selbst einen Gefallen, und überlege JETZT wann du morgen die Übungen machen möchtest. Stelle Dir vielleicht einen Wecker, trage eine Erinnerung in Outlook ein oder schreib dir einen Post-It. Was auch immer für dich am besten ist.

Es gibt keine "perfekte" Zeit zum üben, die für alle Menschen funktioniert. Du musst selbst "deine perfekte Zeit" finden. Nach meiner Erfahrung könnte das einer von diesen Zeitpunkten sein :

- Stehe 15 Minuten früher auf und starte deinen Tag mit den Übungen ODER
- Kombiniere deine Mittagspause mit den Übungen ODER
- Beende deinen Arbeitstag mit den Übungen ODER
- Übe direkt vor dem Einschlafen.

Du musst ausprobieren was dir am besten gefällt. Und dann bleibe bei diesem Zeitpunkt, damit es in deine Routine übergeht. (Übungen dann zu machen "wann es passt", geht selten gut!).

Zur Hilfe hast du hier ein Übungsprotokoll zum Ausdrucken und Ausfüllen im Laufe des Kurses: ÜBUNGSPROTOKOLL

Wenn du während des Kurses Fragen hast, kannst du mich selbstverständlich jederzeit kontaktieren: lotte@vollerelan.de

Ich wünsche dir ganz viel Spaß und viel Erfolg mit deinem ComGrat-Kurs.

Lotte

Lotte Bock
Germaniastraße 93
47800 Krefeld
Deutschland

Mit einem Klick auf den folgenden Link erhältst Du eine aktuelle Selbstauskunft über die über Dich gespeicherten Daten.

Klicken Sie auf den folgenden Link, um Ihre E-Mail-Adresse zu ändern.

Möchtest Du von mir keine E-Mails mehr erhalten? Dann kannst du dich mit nur einem Klick sicher abmelden.

Translation: Introduction Mail

Welcome to the four-week course

Welcome to the four-week mindfulness course ‘Stress-free through your studies’. Mindfulness has many facets and in this course we will mainly deal with the part: emotional skills, empathy, compassion and gratitude.

Tomorrow it can start!

A few tips for the implementation I give you in advance in this video.

VIDEO: Einführung für Studierende zum vierwöchigen Kurs: Stressfrei durchs Studium

If you want to know how empathy, compassion and gratitude are related to emotional skills and emotional intelligence, feel free to watch this video first. Here I explain very briefly what it is about.

VIDEO: EInführung EQ Studierende

STARTING TOMORROW!

You will receive the first email of the course first thing tomorrow morning. Each email contains a short video to inspire you for the day and a video with a meditation. In the evening, you will receive another email reminding you to fill out your gratitude journal.

IMPORTANT NOTES BEFORE THE COURSE:

The daily programme takes no more than 15-20 minutes. That's not much, but you'll find that making time for it is probably going to be the biggest challenge for you.

So do yourself a favour and decide NOW when you want to do the exercises tomorrow. Consider setting an alarm, adding a reminder to your Outlook calendar, or writing a post-it note. Whatever works best for you.

There is no ‘perfect’ time to practice that works for everyone. You have to find ‘your perfect time’ yourself. In my experience, it could be one of these times:

- Get up 15 minutes earlier and start your day with the exercises OR
- combine your lunch break with the exercises OR
- end your working day with the exercises OR

- do them right before you go to sleep.

You have to try out what you like best. And then stick to that point in time so that it becomes part of your routine. (Doing the exercises ‘when it suits’ rarely works!).

To help you, you have an exercise protocol here that you can print out and fill out during the course: EXERCISE PROTOCOL

If you have any questions during the course, you can of course contact me at any time: lotte@vollerelan.de

I wish you lots of fun and much success with your ComGrat course.

Lotte

Lotte Bock

Germaniastraße 93

47800 Krefeld

Germany

By clicking on the following link, you will receive up-to-date information about the data stored about you.

Click on the following link to change your email address.

Do you no longer wish to receive emails from me? Then you can unsubscribe securely with just one click.

Example of a daily E-Mail (German Version)

Stressfrei durch das Studium
# - Tag 20 -

Wusstest du, dass das Erlebnis, sich für jemand anderen zu freuen auch als "altruistische Freude" bezeichnet wird und uns zeigt, dass wir soziale Wesen sind? Aus der Evolution haben wir gelernt, dass wir andere brauchen und dass der Erfolg "des Jägers" eine Voraussetzung für den Erfolg "des Hüters" und vice versa ist. Heutzutage ist eher erstrebenswert, "selbstständig und unabhängig" zu sein, aber in Wirklichkeit ist das Abhängig-Sein eine unserer größten Stärken: Wir sind ALLE voneinander abhängig.
Das Bedürfnis nach Zugehörigkeit und Verbundenheit ist universell bei uns Menschen; wir kommen zusammen in Städten, erweitern unsere Beziehungen auf jeglichen sozialen Netzwerken und Gruppen und genießen die Arbeit aller anderen Menschen, um unseren Lebensstandard aufrechtzuerhalten. Und das ist nicht alles. Wenn wir uns verbunden und zugehörig fühlen, dann wird ein "Glückshormon" namens Oxytocin freigesetzt, und wir fühlen uns einfach gut.
In einer Studie wurde festgestellt, dass Empathie dieses Hormon auslöst. D.h., wenn wir lernen, empathischer zu sein, tut dies nicht nur unseren Mitmenschen gut, sondern trägt auch direkt zu unserem eigenen Wohlbefinden bei.

Empathy toward Strangers Triggers Oxytocin Release and Subsequent Generosity

---

ALLE MEDITATIONEN

Du kannst nun selbst entscheiden, welche Meditation, du morgens machen möchtest. Entweder fängst du wieder bei der ersten an oder du suchst dir eine aus der Liste aus.

Freundlichkeit und das innere Lächeln	Freundlichkeit und ein Wohltäter	Freundlichkeit und ein Familienmitglied
Freundlichkeit und ein Freund	Freundlichkeit und alle Menschen	Freundlichkeit und Sicherheit
Freundlichkeit und Gesundheit	Freundlichkeit und Dankbarkeit	Freundlichkeit und Leichtigkeit
Freundlichkeit für eine geliebte Person	Freundlichkeit für eine neutrale Person	Freundlichkeit für eine schwierige Person
Freundlichkeit für eine Gruppe	Freundlichkeits-meditation komplett	

IMPULS: Entspannung

Das Gefühl von Entspannung.

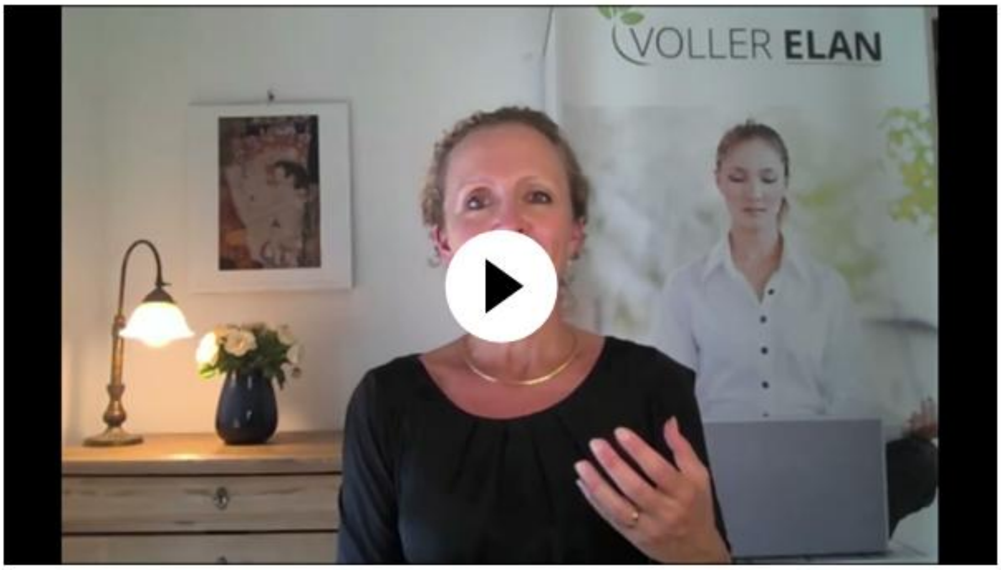

Bis heute Abend!

Lotte

VOLLER ELAN

vollerelan.com

--

Voller Elan
Lotte Bock
Germaniastraße 93
47800 Krefeld
Deutschland

Klicken Sie auf den folgenden Link, um Ihre E-Mail-Adresse zu ändern: https://assets.klicktipp.com/static/email-preview/preview_link.html?type=change_email&lang=de

Möchten Sie von uns keine E-Mails mehr erhalten? Dann können Sie sich mit nur einem Klick sicher abmelden: https://assets.klicktipp.com/static/email-preview/preview_link.html?type=unsubscribe&lang=de

.

Mit einem Klick auf den folgenden Link erhalten Sie eine aktuelle Selbstauskunft über die über Sie gespeicherten Daten: Selbstauskunftslink

Translation: Example of a daily Mail

Stress-free studying

- Day 20 -

Did you know that the experience of being happy for someone else is also called ‘altruistic joy’ and shows us that we are social beings? From evolution, we have learned that we need others and that the success of ‘the hunter’ is a prerequisite for the success of ‘the keeper’ and vice versa. Nowadays, it is more desirable to be ‘independent and self-sufficient’, but in reality, being dependent is one of our greatest strengths: we are ALL dependent on each other.

The need for belonging and connection is universal for us humans; we come together in cities, expand our relationships to include all kinds of social networks and groups, and enjoy the work of other people to maintain our standard of living. And that's not all. When we feel connected and a sense of belonging, a ‘happy hormone’ called oxytocin is released, and we simply feel good.

In one study, empathy was found to trigger this hormone. This means that when we learn to be more empathetic, it not only benefits those around us, but also contributes directly to our own well-being.

Empathy toward Strangers Triggers Oxytocin Release and Subsequent Generosity

ALL MEDITATIONS
(Example of one meditation: vollerelan.de/wp-content/uploads/2022/09/Meditation-lacheln.mp4)

You can now decide for yourself which meditation you would like to do in the morning. Either start again with the first one or choose one from the list.

IMPULSE: Relaxation

VIDEO: Impuls - Entspannung wahrnehmen

The feeling of relaxation.

See you tonight!

Lotte

Example of the Evening Mail (German Version)

DANKBARKEITSTAGEBUCH

Es ist Zeit dein Dankbarkeitstagebuch auszufüllen. Falls du eine kurze Anleitung brauchst findest du drei kurze Dankbarkeits-Meditationen hier. Such dir eine aus und fülle danach das Tagebuch aus.

Einführung für das Dankbarkeitstagebuch - 2 Minuten -

Einführung für das Dankbarkeitstagebuch - 5 Minuten -

Einführung für das Dankbarkeitstagebuch - 7 Minuten -

Tipps fürs Ausfüllen

Wann? Am besten suchst du dir eine bestimme Zeit, in der du in dein **Dankbarkeitsbuch** schreibst. Manch einer schreibt am liebsten abends, während ein anderer gerne morgens darüber nachdenkt, wofür er oder sie dankbar ist. **Ich empfehle dir, die Zeit Abends zu finden** aber wichtig ist nur, dass du dir die Zeit zum Schreiben täglich nimmst. Schon bald wird es zur Gewohnheit und zum Teil deiner täglichen Routine.

Wo? Als Basis für das Dankbarkeitstagebuch reichen ein **Notizblock** oder ein paar Seiten Druckerpapier. Du kannst auch die **App** GRATITUDE im App-Store oder Google Play runterladen und sie nutzen.

Wie? Du überdenkst einfach den Tag und erinnerst dich an Dinge, Menschen oder Umstände, für die du dankbar bist.... oder sehr zufrieden bist. Unten findest du drei kurze Meditationen, die dir helfen können.

Was? Es muss nichts weltbewegend sein oder besonders tiefgründig. Ein freundliches Gesicht, ein nettes Gespräch, ein leckeren Kaffee... auch die kleine Dinge können wir genießen. Es geht ums Gefühl von Dankbarkeit oder Zufriedenheit.

Hier findest du drei kurze Meditationen, die dich für den täglichen Eintrag in dein Dankbarkeitstagebuch vorbereiten können. Anschließend kannst du dein Dankbarkeitstagebuch nehmen, das heutige Datum notieren und deine Punkte notieren.

Lotte Bock
Germaniastraße 93
47800 Krefeld
Deutschland

Mit einem Klick auf den folgenden Link erhältst Du eine aktuelle Selbstauskunft über die über Dich gespeicherten Daten.

Klicken Sie auf den folgenden Link, um Ihre E-Mail-Adresse zu ändern.

Möchtest Du von mir keine E-Mails mehr erhalten? Dann kannst du dich mit nur einem Klick sicher abmelden.

Translation: Evening Mail

GRATITUDE DIARY

It's time to fill out your gratitude diary. If you need a brief introduction, you can find three short gratitude meditations here. Choose one and then fill out the diary.

THREE VIDEOS OF EITHER 2, 5 or 7 MINUTES AS WARM UP FOR THE GRATITUDE ENTRY

vollerelan.de/wp-content/uploads/2022/06/Dankbarkeit-5-Min-FINAL.mp4

Tips for filling it out

When? It's best to find a specific time to write in your **gratitude journal**. Some people prefer to write in the evening, while others like to reflect on what they are grateful for in the morning. **I recommend finding the time in the evening,** but the important thing is to take the time to write every day. Soon it will become a habit and part of your daily routine.

Where? A **notepad** or a few pages of printer paper are enough as a basis for the gratitude diary. You can also download the GRATITUDE **app** from the App Store or Google Play and use that.

How? You simply reflect on the day and remember things, people or circumstances for which you are grateful... or very satisfied. You will find three short meditations below that may help you.

What? It doesn't have to be anything earth-shattering or particularly profound. A friendly face, a nice conversation, a delicious coffee... we can enjoy even the little things. It's about feeling grateful or content.

Here are three short meditations that can prepare you for your daily entry in your gratitude journal. Then you can take your gratitude journal, write down today's date and write down your items.

Lotte Bock
