# Supplementary material for: Targeted mindfulness and self-compassion improve long-term stress reduction in distance learning students: a randomized trial
Source: Front Psychol. 2026 Jan 14;16:1678094. doi: 10.3389/fpsyg.2025.1678094 (PMC12847346; doi:10.3389/fpsyg.2025.1678094)
Supplement: Supplementary file 2 [file Data_Sheet_2.pdf]

Examples of E-Mails from the Mind2Full Course

Introduction Mail (German Version)

Willkommen zum vierwöchigen Kurs

Willkommen!

Ich freue mich sehr, dass du dich für den Kurs entschieden hast.

Hast du schon den Fragebogen ausgefüllt?

Bald kann es auch schon losgehen. Ein paar Tipps für die Durchführung gebe ich dir vorab in diesem Video.

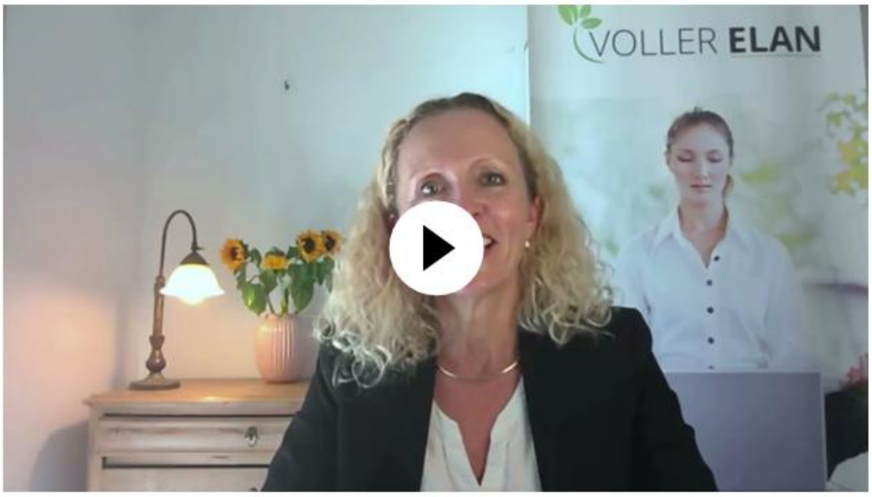

Wenn du noch keine Ahnung von **Achtsamkeit** hast, kannst du dir gerne vorher dieses Video anschauen. Hier erkläre ich ganz kurz, worum es geht.

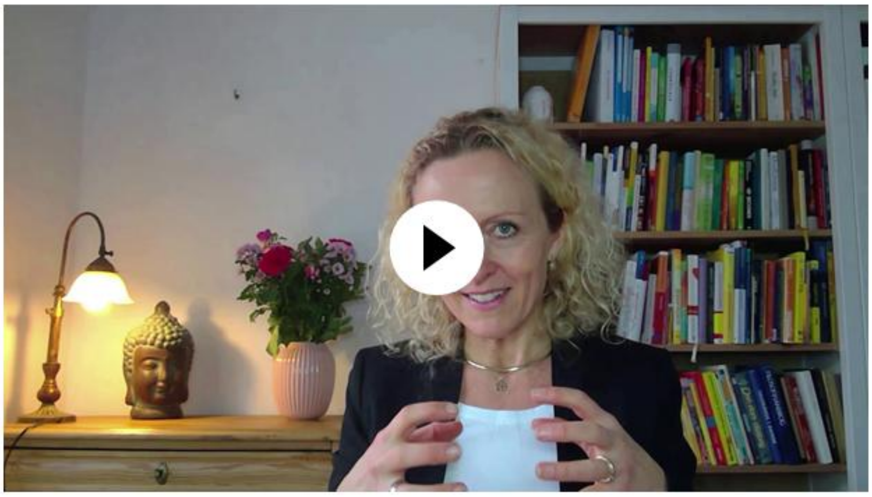

MORGEN GEHT ES LOS!

Schon morgen früh bekommst du die erste E-Mail des Kurses. In jeder E-Mail erhältst du ein Video mit einem kleinen Impuls für den Tag und ein Video mit einer Meditation. Abends bekommst du noch eine E-Mail mit einer Erinnerung an das Ausfüllen des Dankbarkeitstagebuches.

WICHTIGE HINWEISE VOR DEM KURS:

Das tägliche Programm dauert nicht mehr als 15-20 Minuten. Das ist nicht viel, aber du wirst feststellen, dass wahrscheinlich genau hierin die größte Herausforderung für dich bestehen wird, diese Zeit freizuräumen.

Also, tue dir selbst einen Gefallen, und überlege JETZT wann du morgen die Übungen machen möchtest. Stelle Dir vielleicht einen Wecker, trage eine Erinnerung in Outlook ein oder schreib dir einen Post-It. Was auch immer für dich am besten ist.

Es gibt keine "perfekte" Zeit zum üben, die für alle Menschen funktioniert. Du musst selbst "deine perfekte Zeit" finden. Nach meiner Erfahrung könnte das einer von diesen Zeitpunkten sein :

- Stehe 15 Minuten früher auf und starte deinen Tag mit den Übungen ODER
- Kombiniere deine Mittagspause mit den Übungen ODER
- Beende deinen Arbeitstag mit den Übungen ODER
- Übe direkt vor dem Einschlafen.

Du musst ausprobieren was dir am besten gefällt. Und dann bleibe bei diesem Zeitpunkt, damit es in deine Routine übergeht. (Übungen dann zu machen "wann es passt", geht selten gut!).

Zur Hilfe hast du hier ein Übungsprotokoll zum Ausdrucken und Ausfüllen im Laufe des Kurses: ÜBUNGSPROTOKOLL

Wenn du während des Kurses Fragen hast, kannst du mich selbstverständlich jederzeit kontaktieren: lotte@vollerelan.de

Ich wünsche dir ganz viel Spaß und viel Erfolg mit deinem Kurs.
Lotte

Lotte Bock
Germaniastraße 93
47800 Krefeld
Deutschland

Mit einem Klick auf den folgenden Link erhältst Du eine aktuelle Selbstauskunft über die über Dich gespeicherten Daten.

Klicken Sie auf den folgenden Link, um Ihre E-Mail-Adresse zu ändern.

Möchtest Du von mir keine E-Mails mehr erhalten? Dann kannst du dich mit nur einem Klick sicher abmelden.

Translation: Introduction to the Mind2Full Course

Welcome to the four-week course

Welcome!

I'm really happy that you've decided to take this course.

Have you already completed the questionnaire?

We'll be starting soon. This video gives you a few tips for how to do it.

VIDEO: Vierwöchiger Achtsamkeitskurs - eine Einführung

*If you don't know what **mindfulness** is, you can watch this video first. Here I explain very briefly what it is about.*

VIDEO: Was ist Achtsamkeit? Eine kurze Einführung ins Thema.

START TOMORROW!

You will receive the first email of the course tomorrow morning. Each email contains a short video to inspire you for the day and a video with a meditation. In the evening, you will receive another email reminding you to fill out your gratitude journal.

IMPORTANT INFORMATION BEFORE THE COURSE:

The daily programme takes no more than 15-20 minutes. That's not much, but you'll find that making time for it is probably going to be the biggest challenge for you.

So do yourself a favour and decide NOW when you want to do the exercises tomorrow. You could set an alarm, put a reminder in Outlook or write yourself a post-it. Whatever works best for you.

There is no 'perfect' time to practice that works for everyone. You have to find 'your perfect time' yourself. In my experience, it could be one of these times:

- *Get up 15 minutes earlier and start your day with the exercises OR*
- *Combine your lunch break with the exercises OR*
- *End your working day with the exercises OR*
- *Practice right before you go to sleep.*

You have to try out what you like best. And then stick to that point in time so that it becomes part of your routine. (Doing exercises ‘when it suits you’ rarely works!).

To help you, you have an exercise protocol here that you can print out and fill out during the course: EXERCISE PROTOCOL

If you have any questions during the course, you can of course contact me at any time: lotte@vollerelan.de

I wish you lots of fun and much success with your course.

Lotte

Lotte Bock

Germaniastraße 93

47800 Krefeld

Germany

By clicking on the following link, you will receive up-to-date information about the data stored about you.

Click on the following link to change your email address.

Do you no longer wish to receive emails from me? Then you can unsubscribe securely with just one click.

Example of a daily mail from the Mind2Full Course (German Version)

Stressfrei durchs Studium
- Tag 9 -

Liebe/r Kursteilnehmer/-in,

kennst du dieses Szenario: du liegst im Bett, kannst nicht schlafen, weil du nicht aufhören kannst, über etwas zu grübeln. Du sagst zu dir selbst: "Hör doch auf, zu denken" oder "Denk nicht mehr darüber nach", aber das wirkt irgendwie wie eine Einladung für weitere Gedanken, und sie gehen einfach nicht weg. Mit einer kleine Geschickte erkläre ich dir heute, warum das so ist, und natürlich auch, was du dagegen machen kannst.

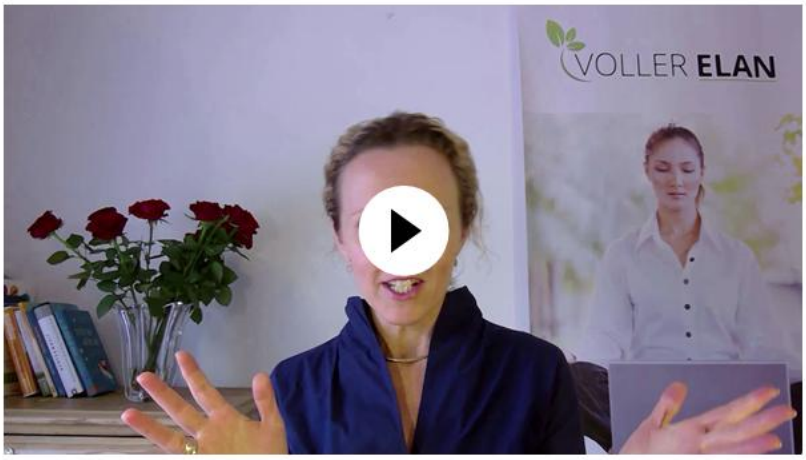

Das Yoga-Programm und die Meditation sind heute kurz und knackig und dauern lediglich jeweils 4 bzw. 5 Minuten.

Viel Spaß!

Lotte

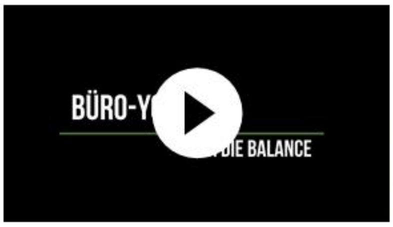

Die Balance - 4 Min.
Dehnungen

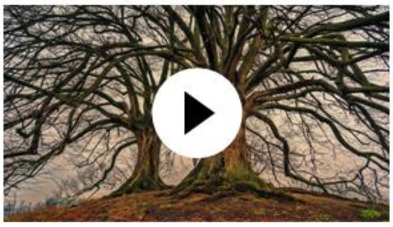

Meditation - 5 Min.
Gedanken einhüllen

VOLLER ELAN

vollerelan.com

--

Stress-free studying

Translation of Daily E-Mail

- Day 9 -

Dear course participant,

are you familiar with this scenario: you are lying in bed, unable to sleep because you cannot stop brooding over something. You say to yourself, 'Stop thinking' or 'Don't think about it anymore,' but somehow that seems like an invitation for more thoughts, and they just won't go away. With a little story, I'll explain to you today why that is, and of course what you can do about it.

VIDEO: https://www.youtube.com/watch?v=zlb8_mjMoEo&feature=youtu.be

Today's yoga programme and meditation are short and sweet and only take 4 and 5 minutes respectively.

Have fun!

Lotte

The Balance VIDEO - 4 Min. Für die Balance

Meditation VIDEO – 5 Min. Gedanken einhüllen

Lotte Bock

Germaniastraße 93

47800 Krefeld

Germany

Click on the following link to change your email address: https://assets.klicktipp.com/static/email-preview/preview_link.html?type=change_email&lang=de

Do you no longer wish to receive e-mails from us? Then you can unsubscribe securely with just one click: https://assets.klicktipp.com/static/email-preview/preview_link.html?type=unsubscribe&lang=de

By clicking on the following link, you will receive an up-to-date self-disclosure of the data stored about you: self-disclosure link
